# Involvement of Igf1r in Bronchiolar Epithelial Regeneration: Role during Repair Kinetics after Selective Club Cell Ablation

**DOI:** 10.1371/journal.pone.0166388

**Published:** 2016-11-18

**Authors:** Icíar P. López, Sergio Piñeiro-Hermida, Rosete S. Pais, Raquel Torrens, Andreas Hoeflich, José G. Pichel

**Affiliations:** 1 Centro de Investigación Biomédica de la Rioja (CIBIR), Fundación Rioja Salud, Logroño, Spain; 2 Institute of Genome Biology, Leibniz-Institute for Farm Animal Biology (FBN), Dummerstorf, Germany; Children's Hospital of Los Angeles, UNITED STATES

## Abstract

Regeneration of lung epithelium is vital for maintaining airway function and integrity. An imbalance between epithelial damage and repair is at the basis of numerous chronic lung diseases such as asthma, COPD, pulmonary fibrosis and lung cancer. IGF (Insulin-like Growth Factors) signaling has been associated with most of these respiratory pathologies, although their mechanisms of action in this tissue remain poorly understood. Expression profiles analyses of IGF system genes performed in mouse lung support their functional implication in pulmonary ontogeny. Immuno-localization revealed high expression levels of Igf1r (Insulin-like Growth Factor 1 Receptor) in lung epithelial cells, alveolar macrophages and smooth muscle. To further understand the role of Igf1r in pulmonary homeostasis, two distinct lung epithelial-specific *Igf1r* mutant mice were generated and studied. The lack of *Igf1r* disturbed airway epithelial differentiation in adult mice, and revealed enhanced proliferation and altered morphology in distal airway club cells. During recovery after naphthalene-induced club cell injury, the kinetics of terminal bronchiolar epithelium regeneration was hindered in *Igf1r* mutants, revealing increased proliferation and delayed differentiation of club and ciliated cells. Amid airway restoration, lungs of *Igf1r* deficient mice showed increased levels of *Igf1*, *Insr*, *Igfbp3* and epithelial precursor markers, reduced amounts of Scgb1a1 protein, and alterations in IGF signaling mediators. These results support the role of Igf1r in controlling the kinetics of cell proliferation and differentiation during pulmonary airway epithelial regeneration after injury.

## Introduction

Adult resident stem or progenitor cells are implicated in both homeostatic tissue maintenance and functional restoration after injury in many organs, including the lung. Following postnatal growth, the lung reaches a steady state in which epithelial turnover is low. However, airway epithelial cells are constantly exposed to and damaged by potential toxic agents and pathogens in the environment, and their subsequent regeneration is a vital process in helping maintain the function and integrity of the lungs. Furthermore, many respiratory disorders, such as asthma, COPD and pulmonary fibrosis, are the consequence of inefficient repair of respiratory epithelial injury and inadequate resolution of airway inflammation [reviewed in [1]].

Lung epithelial composition varies along the proximal-distal axis. In the mouse, interlobar airways (bronchioles) consist mainly of a mixture of secretory non-ciliated cells, club cells, and ciliated cells interspersed with clusters of neuroendocrine (NE) cells. Distally, the alveolar epithelium consists of type 2 (AEC2) and type 1 (AEC1) pneumocytes [1, 2]. Club cells are the predominant cell type in distal bronchioles. They specifically express the secreted protein Scgb1a1/CCSP/CC10, serve as a defense barrier, and additionally show anti-inflammatory, immune-modulating and anti-tumorigenic roles in the lung [3, 4]. Following injury to mouse bronchioles, club cells can both self-renew and give rise to new ciliated cells [5–7]. Different laboratories have used naphthalene treatment in experimental animals to model airway epithelial injury and sluggish recovery to bring knowledge to the field [6, 8–10]. After naphthalene administration, most of the club cells die. This is because they express cytochrome P4502F2 (encoded by *Cyp2f2*) that converts naphthalene into toxic epoxides that leadthem to cell death. Injured and necrotic club cells are replaced by other airway epithelial cells, which undergo dynamic changes in cell migration, proliferation, and differentiation in an attempt to maintain the permeability barrier of the epithelium [9, 11, 12]. The best candidates for airway epithelial regeneration following naphthalene injury are surviving club cells located near neuroendocrine bodies (NEBs) and at the bronchioalveolar duct junction (BADJ), the so called Clara variant cells, which are both necessary and sufficient for restoring bronchiolar epithelium [6, 7, 13]. Despite of some advances made in the last decade, identification of molecular mechanisms involved in this epithelial regenerative process are still poorly understood.

IGF1R (Insulin-like Growth Factor1 Receptor) is a membrane bound tyrosine kinase receptor that is activated by the binding of its two major cognate ligands, the insulin-like growth factors (IGF1 and IGF2). IGF2 also interacts with a second receptor (IGF2R/M6P-R) that reduces IGF2 signaling through lysosomal degradation. During pre- and postnatal development and in the adult, IGF ligands and receptor expression are tightly regulated in a cell type-specific and spatiotemporal manner. In addition, six IGF binding proteins (IGFBPs 1–6) tightly regulate IGF bioavailability to the receptor. Homology between IGF1R and the insulin receptor (INSR) allows IGF signaling through INSR, although with lower affinity; and *vice versa*, insulin (and pro-insulin) can activate IGF1R. Furthermore, IGF1R and INSR can form hybrid receptors, which have a high binding affinity for IGF1, thereby functioning as an IGF1R. Altogether, these proteins constitute the IGF system. Binding of ligands to IGF1R causes activation of various signaling pathways. Canonically the PI3 kinase/Akt, with proven cell survival activity, and the mitogen-activated protein kinases (MAPK), more involved in cell proliferation and differentiation. Additional IGF1R activities include regulation of cell growth, adhesion, migration, metabolism and senescence [reviewed in [14–17]].

IGF signaling is involved in homeostasis of the human lung. Thus, IGFs are implicated in pulmonary vascularization during fetal development, as well as in regeneration and wound lung repair in adulthood, and consequently associated with relevant lung respiratory diseases such as asthma, fibrosis and cancer [18–22]. IGF1R mutations have been identified in humans presenting lung anomalies. One patient with a deletion of the distal long arm of chromosome 15, which includes this receptor, was reported with lung hypoplasia, and more recently, a patient with a homozygous mutation of IGF1R was reported with pulmonary hypertension [23, 24]. Targeted mutations of IGF genes in the mouse indicate that IGF signaling is relevant for lung tissue development, homeostasis and repair. Mutant mice completely lacking *Igf1r* (*Igf1r*^*−/−*^) reach only 45% of normal birth size, are unable to expand their lungs and die shortly after birth [25]. Prenatal *Igf1r*^*-/-*^ embryos reveal conspicuous delayed end-gestational lung maturation characterized by increased cell proliferation and apoptosis [26]. Similarly, we have shown that mice lacking Igf1 are strongly growth retarded and show high postnatal mortality due to hypoplastic lungs marked by increased cellularity and collapsed alveoli. Prenatal lungs from these *Igf1*-null mutants also display high cell proliferation levels as well as epithelial and endothelial abnormalities, reflected by their differential transcriptomic profiles [27–29]. *Ex vivo* stimulation of lung development by IGF1 and IGF2, show that IGF signaling induces vascular and alveolar epithelium maturation in late stages of fetal lung development [29, 30]. Mutant mouse models with Igf1r reduced expression or signaling in adulthood display histopathological alterations in the airway epithelium. Also, increased proliferation rates in the alveolar parenchyma demonstrate better recovery after lung epithelial injury induced by hyperoxia or bleomycin [31–33]. All this scientific evidenceis consistent with IGF signaling being an essential mediator during pulmonary development and homeostasis.

Here we explored the pulmonary expression profiles of IGF system genes during lung development and aging. A detailed characterization of Igf1r protein expression performed at the cellular level in the adult mouse lung revealed high expression levels in lung epithelial cells. To further understand the role of this receptor in the pulmonary epithelium we generated two lung epithelial-specific *Igf1r* gene conditional mutant mouse lines, one generalized and the other restricted to club cells. Lack of *Igf1r* in the airway epithelium disturbs bronchiolar epithelial differentiation in adult mice with a major impact on terminal bronchioles. In terminal bronchioles, the epithelium reveals higher proliferation rates, altered morphology in club cells, and disturbed kinetics of bronchiolar epithelial cell regeneration after a naphthalene challenge. During recovery after club cell injury, bronchiolar epithelial cells of Igf1r-deficient mice show increased proliferation and delayed differentiation of club and ciliated cells, and their lungs reveal altered expression and activation of epithelial-specific markers, Igf genes and Igf signaling mediators. These results support the role of Igf1r in controlling the kinetics of airway epithelial cell proliferation and differentiation during pulmonary airway epithelial regeneration after injury,and revealcellular and molecular mechanisms underlying this process.

## Results

### Spatiotemporal Expression Pattern of IGF System Genes in the Mouse Lung

**Fig 1:**
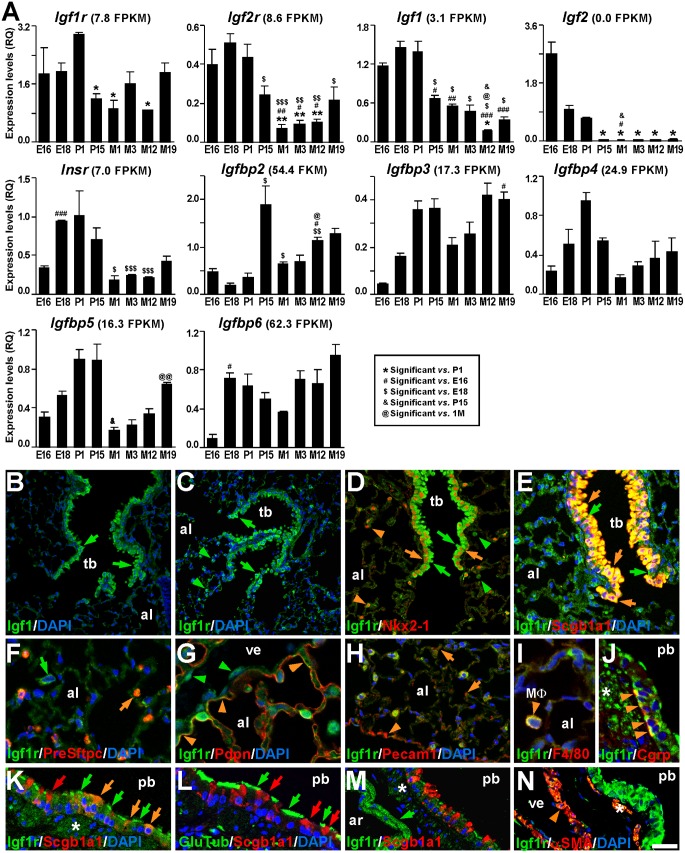
Expression of IGF system genes in the mouse lung. **(A)** Time course graphs of relative mRNA expression of specified IGF system genes analyzed by qRT-PCR during different developmental stages (E16.5, E18.5, P1, P15, M3, M12 and M19) (n = 3). Values in graphs show mean ± SEM. The statistical significance is indicated by specified symbols in the square-bordered legend as follows: one symbol, *p*<0.05; two symbols, *p*<0.01; three symbols, *p*<0.001. E, embryonic day; P, postnatal day; M, postnatal month. FPKM (Fragments Per Kilobase of exon Model per million fragments mapped read) are shown in parenthesis, after the name of the gene and as a reference, as determined by RNA-seq in lungs at the developmental stage M3 (n = 5) (unpublished data). **(B-C)** Immuno-staining for Igf1 (B) and Igf1r (C) (green labeling) in six-month-old lungs. Distal bronchiolar epithelium showed strong staining for both proteins (green arrows). Note that Igf1r was also found scattered throughout the alveolar parenchyma (green arrowheads in C). **(D-N)** Immuno-staining for Igf1r (green labeling), counterstained in red with lung cell-type specific markers and blue with DAPI to visualize nuclei, in three-month-old lungs. (D) All bronchiolar epithelial cells showed co-localization of Igf1r (green arrows) with nuclear Nkx2-1 (orange arrows), and also co-localized with Nkx2-1^+^ AEC2 cells in the alveoli (orange arrowheads). There were Nkx2-1^-^ alveolar cells that additionally stained for Igf1r (green arrowheads). (E) Igf1r strongly stained abundant Scgb1a1^+^ club cells in terminal bronchioles (orange arrows), and in apical cilia of scarce ciliated cells (green arrows). (F) Igf1r stained the cytoplasm of Pre-Sftpc^+^ AEC2 cells (orange arrows), and additional cells in alveolar spaces (green arrow). (G) Igf1r co-stained with Pdpn in areas of the apical membrane in AEC1 cells (orange arrowheads). Note the light staining of Igf1r in vein endothelial cells (green arrowheads). (H) Igf1r co-localized with endothelial Pecam1^+^ cells (orange arrows), more abundantly in capillaries under the pleura (orange arrowhead). (I) Igf1r co-localization with the F4/80^+^ alveolar macrophage marker in cells located in alveolar spaces (orange arrowhead). (J) Igf1r stained Cgrp^+^ neuroendocrine cells (orange arrowheads) in proximal bronchioles. (K) Igf1r staining in Scgb1a1^+^ proximal bronchiole club cells is faint (orange and red arrows), but strong in apical membranes (cilia) of ciliated cells (green arrows). In an adjacent section (L), the Glu-Tubulin (GluTub, a cilium specific marker) stained the same ciliated cells (green arrows) as in K, whereas Scgb1a1 stained club cells (red arrows). (M) Pulmonary artery smooth muscle showed strong staining for Igf1r (green arrow), whereas para-bronchiolar smooth muscle stained fainter (asterisk). (N) αSMA^+^ smooth muscle cells in veins also co-express Igf1r (orange arrowhead), as does the para-bronchiolar smooth muscle (asterisk). All confocal images in B–N are representative samples analyzed from independent experiments. al, alveolus; ar, artery; MΦ, macrophage; pb, proximal bronchiole; tb, terminal bronchiole; ve, vein. Scale bar in N: 32 μm in B; 50 μm in C; 34 μm in D-E; 16.6 μm in F; 12,5 μm in G, I, J; 18 μm in K-L; and 25 μm in M-N.

To define IGF system gene expression profiles during lung development and aging, we performed qRT-PCR for *Igf1r*, *Igf2r*, *Igf1*, *Igf2*, *Insr*, and *Igfbps* using mRNA from mouse lungs at different developmental stages, ranging from embryonic E16.5 to nineteen-month-old mice. Results are shown in Fig 1A. Whereas *Igf1r* showed a sustained constitutive expression, with a moderate peak at P1, *Igf2r*, *Igf1*, *Igf2* and *Insr* expression levels were found to be high during embryonic development, decreasing after P1. *Igf2* mRNA expression was almost undetectable at postnatal stages, as previously described [34, 35]. *Igfbp* genes were found to be constitutively expressed and did not reveal major significant changes, although they all tended to increase their expression during embryonic development up to P1, then decrease after birth and increase again moderately with aging, with the exception of *Igfbp2* which shows an increase at P15 (Fig 1A). *Igfbp1* mRNA expression was undetectable in the lung at all stages tested (data not shown). Absolute mRNA expression in lungs, calculated by transcriptome ultrasequencing of 3 month old normal mice (n = 5) (data submitted to Gene Expression Omnibus, accession number GSE88836), ranged from 62.3 FPKM (Fragments Per Kilobase of exon Model per million mapped reads) [1 FPKM, roughly corresponding to 1 mRNA/average cell [36]] for *Igfbp6*, down to undetectable levels for *Igf2* and *Igfbp1* (Fig 1A). Additionally, RNA-seq data also showed undetectable levels for *Ins1* and *Ins2* genes(data not shown). Compared to the moderate expression of *Igfbps*, levels of *Igf1r*, *Igf2r* and *Insr* receptors and the *Igf1*ligandwere lower. However, in all cases they concur with pulmonary mRNA expression levels of these genes in the adult human lung (http://www.proteinatlas.org) [37].

In order to identify their cellular localization and relative protein expression levels in the adult mouse lung, we performed immunohistochemical staining for Igf1, the unique ligand of the IGF system present in the adult mouse lung, and for Igf1r, the main cell autonomous mediator of Igf1 action. Igf1 was found to be highly expressed in bronchiolar epithelial cells (Fig 1B), as well as in the smooth muscle of the pulmonary artery (data not shown). Similarly, Igf1r also showed intense staining in bronchiolar epithelial cells, with an additional punctuated pattern in the alveolar area, and a fainter ubiquitous staining throughout the lung (Fig 1C). To further identify Igf1r cellular localization, we performed immunohistochemical co-staining for Igf1r with specific markers for different pulmonary cell types(1), followed by confocal microscopy analysis. We found differences in staining patterns and intensity among different cell compartments and cell types (Fig 1D–1N). In the distal lung, Igf1r strongly stained the cytoplasm of all Nkx2-1^+^ epithelial cells, including airway epithelial cells and AEC2 in the alveolar parenchyma (Fig 1D), and strictly co-localized in pattern and intensity with Scgb1a1/CC10/CCSP in the cytoplasm of all terminal bronchiolar club cells (Fig 1E). Igf1r also showed strong staining in the luminal side of ciliated cells in distal airways (Fig 1E), as demonstrated by its co-localization in adjacent sections with detyrosinated αTubulin (Glu-Tubulin), a marker for rodent lung ciliated cells [38] (data not shown). In the alveolar parenchyma, Igf1r co-localized with the surfactant protein C precursor (Pre-Sftpc) in the cytoplasm of AEC2 (Fig 1F). At lower intensity, staining for Igf1r was also found in areas of AEC1 apical membranes, as shown by its partial co-localization with podoplanin (Pdpn) (Fig 1G). Igf1r was additionally found in vascular endothelial cells of small veins (Fig 1G), in certain capillaries (co-localizing with Pecam1, Fig 1H), and in alveolar macrophages, (co-staining with the F4/80 marker) [39](Fig 1I). In proximal airways, Igf1r stained the cytoplasm of both Cgrp^+^ neuroendocrine cells grouped in neuroendocrine bodies (NEB) (Fig 1J) and Scgb1a1^+^ club cells (Fig 1K). It is important to note that Igf1r staining intensity in club cells of proximal bronchioles was much fainter than that found in terminal bronchioles, and even some Scgb1a1^+^ cells were found almost devoid of Igf1r labeling (Fig 1K *vs*. 1E). The abundant ciliated cells in proximal aiways also expressed high levels of Igf1r in their luminal side, coinciding with the cilia (Fig 1K), as shown by Glu-Tubulin staining in an adjacent tissue section (Fig 1L). Finally, Igf1r was identified in different types of pulmonary smooth muscle, showing the strongest stain in the pulmonary artery (Fig 1M). With less intensity and presenting a scattered pattern, Igf1r also stained the smooth muscle of pulmonary veins and the parabronchiolar smooth muscle, co-localizing with the alpha-smooth muscle actin (αSMA) marker (Fig 1N). In summary, although Igf1r protein is present throughout the entire adult mouse lung, higher levels were found in epithelial cells, alveolar macrophages, and the smooth muscle. Similar expression patterns were previously described in both the prenatal mouse lung and the adult human lung [29, 37].

### Generation of Mutant Mice with a Lung Epithelium-Specific Deletion of *Igf1r*

**Fig 2:**
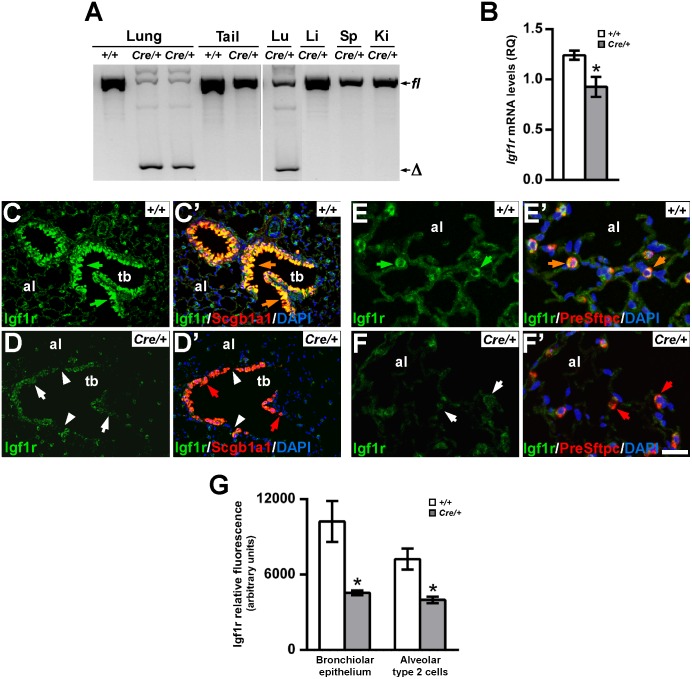
Specific *Igf1r* gene deletion and reduced expression in lungs of adult *Nkx2-1-Cre; Igf1r*^*fl/fl*^ double transgenic mice. **(A)** PCR assays to determine the presence of the deleted allele (Δ) using F3/R1 primers on genomic DNA obtained from different organs of *Igf1r*^*fl/fl*^(*+/+*) and *Nkx2-1-Cre; Igf1r*^*fl/fl*^ (*Cre/+*) mice. The 491 bp fragment of the deleted form *(Δ*) is present in lungs of *Cre/+* animals in detriment of the 1300 bp floxed *(fl)* allele (S1 Fig, panels C-D), but absent in all other tissues tested, independently of the genotype. Ki, kidney; Li, liver; Lu, lung; Sp, spleen. **(B)***Igf1r* mRNA relative expression levels analyzed by qRT-PCR in lungs of *Igf1r*^*fl/fl*^ (+/+) (n = 5) and *Nkx2-1-Cre; Igf1r*^*fl/fl*^ (*Cre/+*) (n = 3) mice.**(C-F')** Immuno-staining for Igf1r in lungs of *Igf1r*^*fl/fl*^ (+/+) (C, C', E, E’) and *Nkx2-1-Cre; Igf1r*^*fl/fl*^ (*Cre/+*) (D, D', F, F'). Sections were co-stained either with Scgb1a1 to identify club cells in terminal bronchioles (C’-D’, orange/red), or with Pre-Sftpc to identify AEC2 in alveoli (E’-F’, orange/red). Note the lack of Igf1r green staining in both terminal bronchioles and AEC2 epithelial cells on *Cre/+* samples (white arrows in D and F, respectively), which do not co-stain with their specific markers, Scgb1a1 and PreSftpc, respectively (red arrows in D’ and F’). Remnant green staining in epithelial cell types could be due to either epithelial cells nonspecific autofluorescence or cells with undeleted *Igf1r* gene (mosaic deletion). Note the altered morphology and interruptions (white arrowheads in D-D’) in the epithelium of terminal bronchioles in the mutants (D-D’), compared to control mice (C-C’). al, alveolus; tb, terminal bronchiole. Scale bar in F': 50 μm in C-D'; 17 μm in E-F’.**(G)** Quantification of Igf1r relative fluorescence in bronchiolar and alveolar type 2 epithelial cells (n = 4 per genotype).*+/+*, *Igf1r*^*fl/fl*^ and *Cre*/+, *Nkx2-1-Cre; Igf1r*^*fl/fl*^genotypes. Values in graphs show mean ± SEM. *, p<0.05.

**Fig 3:**
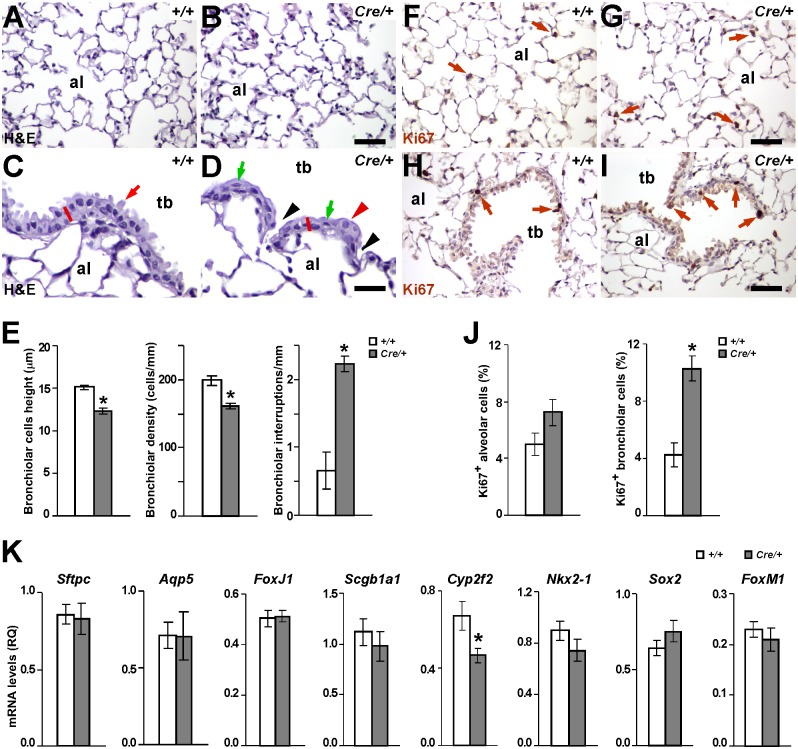
Alterations in histology and cell proliferation in lungs of *Nkx2-1-Cre; Igf1r*^*fl/fl*^ mutant mice. **(A-D)** Representative H&E staining of lung alveoli (A-B) and terminal bronchioles (C-D) sections obtained from *Igf1r*^*fl/fl*^ (*+/+*) (A, C) and *Nkx2-1-Cre; Igf1r*^*fl/fl*^(*Cre*/+) (B, D) mice. Note that in contrast to the normal alveolar parenchyma found in double transgenic mice (B), their bronchiolar epithelium shows histological alterations (D), including: lack of protruding club cells cupules (abundant in controls; red arrow in C), epithelial flattening (see shortening in red segments), lower cell density, abundance of interruptions (black arrowheads), presence of aberrant ellipsoid nuclei (green arrows) and big pale rounded cells (red arrowhead). al, alveolus; tb, terminal bronchiole. *+/+*, *Igf1r*^*fl/fl*^ and *Cre*/+, *Nkx2-1-Cre; Igf1r*^*fl/fl*^genotypes. Scale bars: 50 μm. **(E)** Graphical representations after quantification of bronchiolar epithelium alterations (cell height, cell density, and interruptions in epithelium continuity) in both genotypes (n = 4). **(F-I)** Representative images of Ki67 immuno-staining in brown (brown arrows) in alveolar parenchyma (F-G) and terminal bronchioles (H-I) of *Igf1r*^*fl/fl*^ (*+/+*) (F and H) and *Nkx2-1-Cre; Igf1r*^*fl/fl*^ (*Cre*/+) (G and I) mice. Note the increased presence of Ki67^+^ cells in the bronchiolar epithelium of *Nkx2-1-Cre; Igf1r*^*fl/fl*^ mutant mice. **(J)** Graphical representation of Ki67^+^ based proliferation rates (n = 4). **(K)** mRNA expression levels of genes analyzed by qRT-PCR in total lung homogenates from *Igf1r*^*fl/fl*^ (*+/+*) (n = 5) and *Nkx2-1-Cre; Igf1r*^*fl/fl*^ (*Cre*/+) (n = 3) three month old mice. Values in graphs show mean ± SEM. * p<0.05.

Considering the high expression levels of Igf1r in lung epithelial cells, we generated and analyzed *Nkx2-1-Cre; Igf1r*^*fl/fl*^ and *Scgb1a1-Cre; Igf1r*^*fl/fl*^ transgenic mouse lines, in order to determine Igf1r function in the pulmonary epithelium. *Nkx2-1-Cre; Igf1r*^*fl/fl*^ mice specifically delete the floxed *Igf1r* sequences throughout the mouse lung epithelium driven by the *Nkx2-1-Cre* transgene [40, 41], and *Scgb1a1-Cre; Igf1r*^*fl/fl*^ double transgenics selectively disrupt *Igf1r* gene in bronchiolar epithelial club cells directed by the *Scgb1a1-Cre* construct [42] (S1 Fig), panels A-C. Both mutant mouse lines were fertile and showed apparently normal phenotypes, although we noticed significant overweig in *Nkx2-1-Cre; Igf1r*^*fl/fl*^ mice after puberty when compared with *Igf1r*^*fl/fl*^ normal littermates (data not shown), condition that could be due to the lack of Igf1r in thyroid cells as a consequence of Cre expression in these cells driven by the *Nkx2-1* promoter, as previously reported [40, 43]. Accordingly, mice with a homozygous thyroidal null mutation in the Igf1r locus also showed increased body weight [44]. A PCR-based analysis of genomic DNA, obtained from a variety of tissues to determine the Cre-mediated deletion in the *Igf1r* gene (S1 Fig, panels D-G), demonstrated that it specifically occurs in the adult lungs of both double transgenic mutants, but with much higher efficiency in *Nkx2-1-Cre; Igf1r*^*fl/fl*^ (Fig 2A) than in *Scgb1a1-Cre; Igf1r*^*fl/fl*^ mice (S1 Fig, panels F-G). qRT-PCR in RNA from adult lungs of both *Igf1r* conditional mutants revealed a significant depletion of *Igf1r* mRNA levels in *Nkx2-1-Cre; Igf1r*^*fl/fl*^ lungs (Fig 2B), an effect not observed in *Scgb1a1-Cre; Igf1r*^*fl/fl*^ mice (1.607 RQ in *Igf1r*^*fl/fl*^*vs*. 1.115 RQ in *Scgb1a1-Cre; Igf1r*^*fl/fl*^, p = 0.275; n = 3 per genotype). We did not find reduced levels of *Igf1r* mRNA in lungs obtained from P15 (two weeks old) *Nkx2-1-Cre; Igf1r*^*fl/fl*^ mice (n = 5; data no shown). We next performed *Igf1r* immuno-staining in adult lungs of both transgenic lines to determine the lack of Igf1r protein expression in lung epithelial cells. We noticed a strong depletion in Igf1r^+^ cells and Igf1r relative fluorescence in the terminal bronchiolar epithelium of *Nkx2-1-Cre; Igf1r*^*fl/fl*^ mice (Fig 2C and 2D’, and fluorescence quantification in Fig 2G), which was not as uniform and consistent in *Scgb1a1-Cre; Igf1r*^*fl/fl*^ mutants (S2 and S6 Figs, panels A-B). Igf1r staining at different older stages during aging (tested at three, six, twelve and eighteen months of age) in both mutants showed similar results (S2 and S6 Figs, panels A-B, and data not shown). Igf1r deficiency in epithelial cells of terminal bronchioles revealed several consistent alterations in adult *Nkx2-1-Cre; Igf1r*^*fl/fl*^ mutants, including smaller epithelial cell size and a lower proportion of Scgb1a1^+^ club cells (Fig 2D and 2D’). We also noticed a lack of Igf1r expression in the proximal airwayepithelial cells of these mutants, though they were found only in discrete areas and at a low frequency (data not shown). These results indicate that Cre-mediated *Igf1r* deletion occurred in a mosaic pattern in airway epithelial cells, with the best efficiency found in terminal bronchioles, in areas close to BADJs. As expected, *Nkx2-1-Cre; Igf1r*^*fl/fl*^ mice also showed a lack of Igf1rimmunostaining in Pre-Sfptc^+^ AEC2 pneumocytes in alveoli(Fig 2E and 2F’, and fluorescence quantification in Fig 2G), however no evident gross morphological alterations were noticed either in AEC2 cells themselves or in the surrounding alveolar parenchyma (Fig 3A and 3B).

Alterations in Scgb1a1 staining patterns of club cells, including smaller cell size and reduction in the proportion of Scgb1a1^+^ club cells, were much less common, but also present in some terminal bronchioles of adult *Scgb1a1-Cre; Igf1r*^*fl/fl*^ mice (S2 and S6 Figs, panels A-B). These alterations were not found in proximal airways (data not shown). The unreliable patterns found made it impossible to perform a quantitative evaluation of phenotypical changes in *Scgb1a1-Cre; Igf1r*^*fl/fl*^ mice, however they served as a valuable tool to support the epithelial phenotype found in *Nkx2-1-Cre; Igf1r*^*fl/fl*^ mutants.

### *Igf1r* Deletion in Lung Epithelial Cells Causes Histopathological Alterations and Increased Proliferation in Terminal Bronchiolar Epithelium

Phenotypical consequences of *Igf1r* deletion in lung epithelium of *Nkx2-1-Cre; Igf1r*^*fl/fl*^ mice were analyzed by histology. As mentioned, H&E staining did not reveal gross pathological alterations in alveolar parenchyma (Fig 3A and 3B). However, the distal bronchiolar epithelium of these mutants consistently showed morphological changes compared to controls, including lower cell density, flatter cells with elongated nuclei and frequent interruptions of normal columnar organization (Fig 3C and 3D and S3 Fig, panels A-D). Quantification of these histopathological changes are represented in Fig 3E. As expected, we did not find consistent histological alterations in club cells of *Scgb1a1-Cre; Igf1r*^*fl/fl*^ terminal airways (S4 Fig, panels A-B). In addition, it is noteworthy that neither of the two *Igf1r* mutant transgenic mouse lines showed apparent morphological alterations in the lung epithelium when analyzed at early stages of postnatal lung development, including postnatal day (P) 1, P5 and P15 (data not shown).

Considering the assumed pro-mitotic activity of Igf1/Igf1r signaling on epithelial cells and the decreased cell density found in the bronchiolar epithelium of *Nkx2-1-Cre; Igf1r*^*fl/fl*^ mutants, we decided to check if *Igf1r* deficiency in lung epithelial cells could result in reduced cellular proliferation in the lungs of *Nkx2-1-Cre; Igf1r*^*fl/fl*^ mice. Proliferation rates were determined by Ki67 immuno-staining in both pulmonary alveolar parenchyma and epithelial cells of terminal airways of the control and *Igf1r* conditional mutants of this transgenic line. In accordance with the histological data mentioned above (Fig 3A and 3B), we did not find significant differences in cell proliferation rates between genotypes in alveolar areas (Fig 3F, 3G and 3J, left panel). Paradoxically, despite the reduced epithelial cell density found in distal bronchioles of *Nkx2-1-Cre; Igf1r*^*fl/fl*^ mutant lungs, their mitotic rate was more than double that of their control littermates (Fig 3H, 3I and 3J, right panel). Determination of cellproliferation by BrdU incorporation analysis between terminal bronchioles of *Scgb1a1-Cre; Igf1r*^*fl/fl*^ and *Igf1r*^*fl/fl*^ control mice did not reveal meaningful differences (S4 Fig, panels I-J).

To determine if the morphological alterations found in terminal airways of *Nkx2-1-Cre; Igf1r*^*fl/fl*^ mutant mice were reflected in the differential expression of epithelial differentiation markers, we analyzed mRNA expression levels of *Sftpc* and *Aqp5*, as specific markers for AEC2 and AEC1 cells, respectively; *FoxJ1* as a marker for ciliated cells; *Scgb1a1* and *Cyp2f2* as markers for club cells; and *Nkx2*.*1*, *Sox2* and *FoxM1* as transcriptional regulators of lung epithelial differentiation [45, 46]. Among them, only *Cyp2f2*, a cytochrome P-450 monooxygenase specific to club cells, showed differential mRNA expression, revealing significantly lower levels in *Nkx2-1-Cre; Igf1r*^*fl/fl*^ mutant lungs (Fig 3K), a result that further supports the role of Igf1r in maintaining club cells entity.

### Induced Expression of IGF Signaling Genes after Club Cell Ablation with Naphthalene

**Fig 4:**
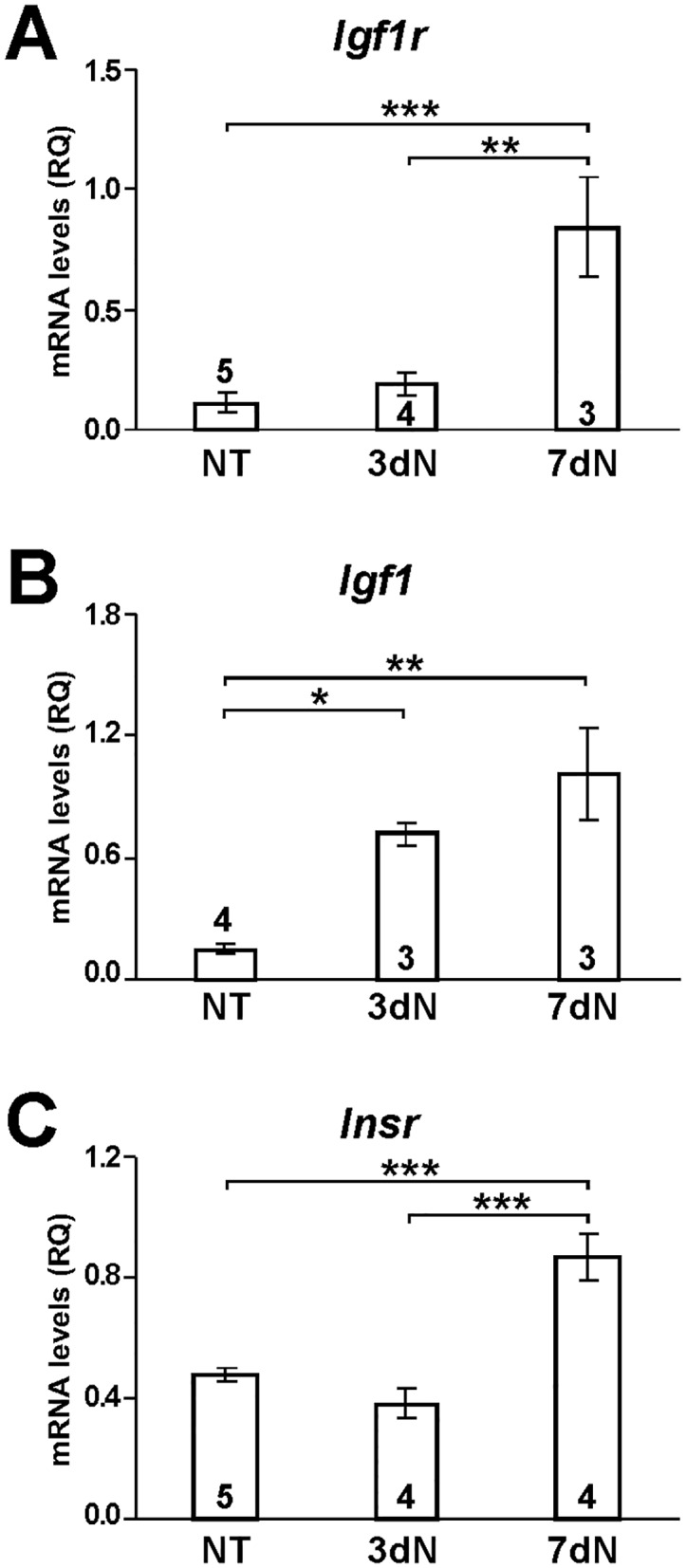
Increased mRNA expression of IGF signaling genes after the naphthalene challenge. qRT-PCR analysis of *Igf1r* (A), *Igf1* (B) and *Insr* (C) mRNA levels before (NT), and three (3dN) and seven (7dN) days after naphthalene administration. Numbers in bars indicate mice analyzed. Values in graphs show mean ± SEM. Data are normalized to 18S RNA expression. RQ: relative quantity. *, *p*<0.05; **, *p*<0.01; ***, *p*<0.001 (Kruskal-Wallis test).

To determine if IGF signaling genes are involved in club cell regeneration, a naphthalene-induced lung injury approach was performed on *Igf1r*^*fl/fl*^ mice. Club cells expressing the cytochrome P-450 monooxygenase Cyp2f2 are the primary targets for the cytotoxicant naphthalene; Cyp2f2 is essential for the bioactivation and toxicity of naphthalene. Changes in mRNA expression of *Igf1r*, *Igf1* and *Insr* were analyzed by qRT-PCR in lungs of non-treated (NT) and naphthalene-treated animals after 3 (3dN) and 7 (7dN) days (Fig 4). Interestingly, whereas *Igf1r* and *Insr* did not show differences in mRNA levels at 3dN (Fig 4A and 4C), their expression was induced at 7dN (more than four times and twice, respectively)*Igf1* mRNA levels were found significantly increased at both stages after the challenge (Fig 4B). Thus, increased expression of these genes after the naphthalene challenge further suggests that IGF signaling is involved in airway club cell regeneration.

### Delayed Cell Differentiation and Sustained Proliferation During Repair of Bronchiolar Epithelium after Club Cell Ablation

**Fig 5:**
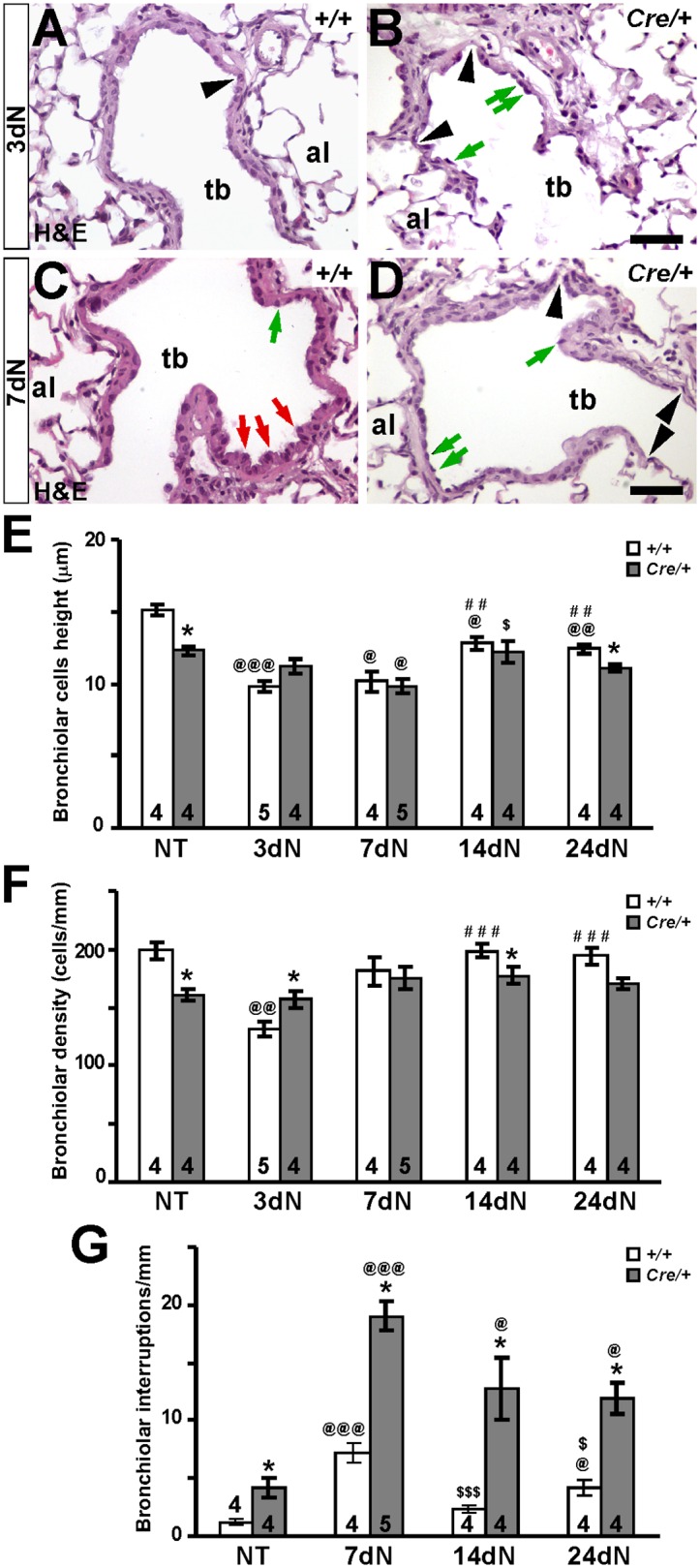
Histopathological changes in terminal bronchioles of *Nkx2-1-Cre; Igf1r*^*fl/fl*^ mutant mice during regeneration after naphthalene challenge. **(A-D)** Representative H&E staining of bronchiole sections at three (3dN) (A-B) and seven (7dN) (C-D) days after the naphthalene treatment analyzed in *Igf1r*^*fl/fl*^ (*+/+*) (A, C) and *Nkx2-1-Cre; Igf1r*^*fl/fl*^(*Cre*/+) (B, D) mice. Red arrows in C point to club cells. Note the increased proportion of bronchiolar epithelial cells with ellipsoid nuclei protruding in the bronchiolar lumen (green arrows) and more abundant interruptions in epithelial continuity (black arrowheads) in double transgenic mutant mice (B, D). al, alveolus; tb, terminal bronchiole. Scale bars: 50 μm. **(E-G)** Quantification of bronchiolar epithelial alterations (See S3 Fig for representative images). Graphics represent changes in bronchiolar cell height (E), cell density (F), and interruptions (G) between genotypes in non-treated (NT), and at three (3dN), seven (7dN), fourteen (14dN) and twenty-four (24dN) days after the naphthalene treatment. *+/+*, *Igf1r*^*fl/fl*^ and *Cre*/+, *Nkx2-1-Cre; Igf1r*^*fl/fl*^genotypes. Numbers in bars indicate mice analyzed. Values in graphs show mean ± SEM. The statistical significance is indicated by specified symbols as follows: *, *+/Crevs*. *+/+* in same condition; @, *vs*. NT of same genotype; #, *vs*. 3dN of same genotype; $, *vs*. 7dN of same genotype; one symbol, *p*<0.05; two symbols, *p*<0.01; three symbols, *p*<0.001.

To assess Igf1r implication in airway epithelial regeneration, we analyzed the capacity of *Igf1r* mutant mice lung epithelium for restoring its integrity at different stages after naphthalene treatment. Epithelial regeneration was monitored at 3dN, 7dN, 14dN and 24dN by H&E histological staining. Representative histopathological alterations in the distal airway epithelium of *Nkx2-1-Cre; Igf1r*^*fl/fl*^ after naphthalene treatment are shown in Fig 5A–5D (3dN and 7dN) and S3 Fig (all stages), and their quantifications are represented in Fig 5E–5G. At 3dN, exfoliation of injured and necrotic bronchiolar epithelial cells left the basement membrane denuded or with only flattened epithelial cells remaining, in both *Igf1r*^*fl/fl*^ and *Nkx2-1-Cre; Igf1r*^*fl/fl*^ mice (Fig 5A and 5B). Although medium cell height was not different at this stage (Fig 5E), epithelial cell density was higher in the mutants (Fig 5F). Remaining naphthalene resistant cells were characterized by showing their nuclei protruding in the bronchiolar lumen (Fig 5B). By 7dN the bronchiolar airway started to show sporadic areas of restoration, with the presence of some domed presumptive club cells in control mice, cell types that were less abundant in *Nkx2-1-Cre; Igf1r*^*fl/fl*^ mutant mice (Fig 5C and 5D). Epithelial interruptions were more abundant in the mutants at this stage (Fig 5G). By 14dN the bronchiolar airway epithelium appeared to be partially restored with areas of denuded basement membrane still visible in control mice (S3 Fig, panel I). This recovery was delayed in the mutant lungs (S3 Fig, panel J), which still showed significantly reduced bronchiolar cell density and an increased presence of epithelial interruptions (Fig 5F and 5G). Finally, regeneration of the airway epithelium at 24dN was observed to be almost complete in *Igf1r*^*fl/fl*^ mice (S3 Fig, panel K), but still with notable alterations in *Nkx2-1-Cre; Igf1r*^*fl/fl*^ mutants, including the presence of extended cell denuded areas (S3 Fig, panel L), and an increased presence of both flat epithelial cells and epithelial interruptions (Fig 5E–5G). At this later stage, all morphological parameters tended to decrease toward levels of NT mice (Fig 5E and 5G). Milder and less consistent histopathology was found scattered in some airways of *Scgb1a1-Cre; Igf1r*^*fl/fl*^ mice analyzed at 3dN, 7dN and 14dN (S4 Fig, panels C-H). As expected, injection of vehicle (corn oil) did not produce any damage in the mouse lung of either genotype (data not shown).

**Fig 6:**
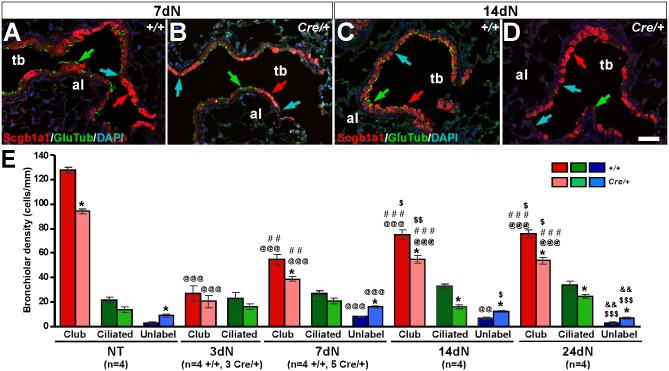
Delayed regeneration of club and ciliated cells in *Nkx2-1-Cre; Igf1r*^*fl/fl*^ terminal bronchioles after naphthalene challenge. **(A-D)** Representative images of immuno-staining to identify club/Scgb1a1^+^ cells (in red) and ciliated/GluTub^+^ (in green) in lungs of *Igf1r*^*fl/fl*^ (+/+) (A, C) and *Nkx2-1-Cre; Igf1r*^*fl/fl*^ (*Cre/+*) (B, D) at 7dN (A-B) and 14dN (C-D) days after naphthalene treatment. Note the lower proportion of club (red arrows) and ciliated cells (green arrows), and the increased presence of non-labeled epithelial cells (blue arrows) in *Cre/+* mutants. (See S5 Fig for representative images). al, alveolus; tb, terminal bronchiole. Scale bar in D: 50 μm. **(E)** Quantification of bronchiolar epithelial cell types labeled in the bronchiolar epithelium following the above-mentioned criteria. *+/+*, *Igf1r*^*fl/fl*^ and *Cre*/+, *Nkx2-1-Cre; Igf1r*^*fl/fl*^genotypes (See S5 Fig for representative images). Values in graphs show mean ± SEM. Unlabel, unlabeled cells with either Scgb1a1 or GluTub. The statistical significance between stages is indicated by specified symbols as follows: *, *+/Crevs*. *+/+* in same condition; @, *vs*. NT of same genotype; #, *vs*. 3dN of same genotype; $, *vs*. 7dN of same genotype; &, *vs*. 14dN of same genotype; one symbol, *p*<0.05; two symbols, *p*<0.01; three symbols, *p*<0.001.

In order to follow the cell fate of club cells during repair after naphthalene injury, double immuno-fluorescent staining for the club cell marker Scgb1a1 and the ciliated cell marker Glu-Tubulin were analyzed by confocal microscopy in terminal bronchioles of *Igf1r*^*fl/fl*^ and *Nkx2-1-Cre; Igf1r*^*fl/fl*^ mice. Results are shown in Fig 6 and S5 Fig. In NT mice, abundant club cells were detected along the bronchiolar airway epithelium of both genotypes, with sporadic presence of ciliated GluTub^+^ and non-labeled (Scgb1a1^-^/GluTub^-^) cells (S5 Fig, panels A-B). Quantification did not revealed significant changes in the mean density of ciliated cells, but Scgb1a1^+^ cells density was reduced. Conversely, unlabeled epithelial cells were more abundant in distal airways of *Nkx2-1-Cre; Igf1r*^*fl/fl*^ NT mutant mice (Fig 6E). At 3dN, the number of Scg1a1^+^ cells was substantially diminished within the residual epithelial cells in terminal bronchioles of both mouse genotypes; these Scgb1a1^+^ remnant cells mainly localized in the BADJs and in additional discrete areas, surrounding presumptive NEBs, where naphthalene-resistant club cell precursors reside (S5 Fig, panels C-D). Injured and necrotic bronchiolar epithelial cells, which had exfoliated into the airway lumen, also stained positively for Scgb1a1 in both genotypes (data not shown). No significant differences in club or ciliated cells counts were found between genotypes at 3dN (Fig 6E). At 7dN, 14dN and 24dN a significantly decreased density of club cells and increased density of unlabeled cells was noticed again in the mutants. Interestingly, the bronchiolar density of ciliated cells was also found to be reduced at 14dN and 24dN (Fig 6 and S5 Fig, panels E-L). Descriptive data obtained by immuno-staining for Scgb1a1 performed in *Scgb1a1-Cre; Igf1r*^*fl/fl*^ mice, also demonstrates the lack of club cells in the epithelium during recovery after naphthalene injury (S6 Fig). Together these results indicate that lack of *Igf1r* in the mouse lung airway epithelium alters cell regeneration in distal airways after naphthalene injury, delaying the differentiation of club and ciliated cells.

**Fig 7:**
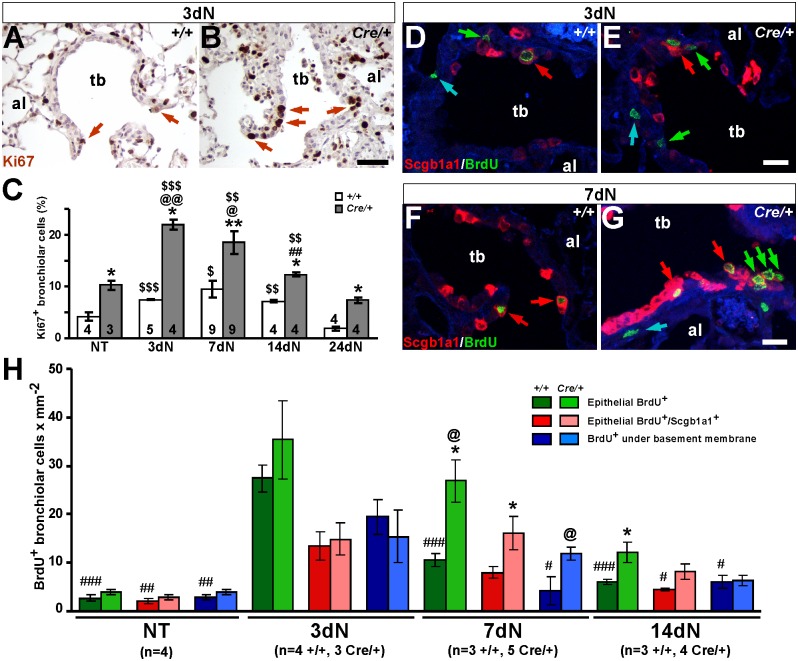
Increased cell proliferation rates in terminal bronchioles of *Nkx2-1-Cre; Igf1r*^*fl/fl*^ mice at different stages during repair after naphthalene challenge. **(A-B)** Representative images of Ki67 immunostaining (brown arrows) in terminal bronchioles of *Igf1r*^*fl/fl*^ (+/+) (A) and *Nkx2-1-Cre; Igf1r*^*fl/fl*^ (*Cre*/+) (B) lungs at 3dN. Note the increased presence of Ki67^+^ in the epithelium of *Cre*/+ mutant mice with respect to *+/+* controls. **(C)** Quantification of Ki67-based proliferation rates in both genotypes (See S7 Fig for representative images). Numbers in bars indicate mice analyzed. **(D-G)** Identification of proliferating cells by anti-BrdU immunostaining (green labeled nuclei) counterstained in red with an anti-Scgb1a1 antibody to mark club cells, allowing for differentiation of three groups of cells: total BrdU^+^ cells in the bronchiolar epithelium (including Scgb1a1^+^ cells) (green arrows), BrdU^+^/Scgb1a1^+^ double labeled cells (red arrows) and BrdU^+^ cells under the epithelial basal membrane (blue arrows) at 3dN (D-E) and 7dN (F-G) in control (*+/+*) (D, F) and mutant (*Cre/+*) (E, G) lungs. al, alveolus; tb, terminal bronchiole. Scale bars: 50 μm in A-B; 25 μm in D-G. **(H)** Quantification of BrdU^+^ cells in terminal bronchioles of control (*+/+*) and mutant (*Cre/+*) mice. The graph represents the quantification of total BrdU+ cells found in the epithelium (green bars), BrdU^+^/Scgb1a1^+^ club epithelial cells (red/pink bars) and in blue bars BrdU+ cells under the basal membrane (up to 12 μm distance) in non-treated (NT), and at three (3dN), seven (7dN) and fourteen (14dN) days after naphthalene treatment (red/pink bars) (see S8 Fig for representative images). Values in graphs show mean ± SEM. The statistical significance between stages is indicated by specified symbols as follows: *, *+/Crevs*. *+/+* in same condition; @, *vs*. NT of same genotype; #, *vs*. 3dN of same genotype; $, *vs*. 24dN of same genotype; one symbol, *p*<0.05; two symbols, *p*<0.01; three symbols, *p*<0.001.

In an attempt to understand this phenotype, cell proliferation and death rates were determined in terminal airways of *Nkx2-1-Cre; Igf1r*^*fl/fl*^ mice following naphthalene treatment. Proliferation analyses by Ki67 immuno-staining are illustrated in Fig 7A and 7B (referring 3dN) and S7 Fig (all stages), and their quantifications shown in Fig 7C. When compared to NT mice, proliferation in *Igf1r*^*fl/fl*^ control mice was greatest at 7dN, progressively decreasing until 24dN, when it reached basal levels (Fig 7C). In the *Nkx2-1-Cre; Igf1r*^*fl/fl*^ mutants it followed a similar profile, however the maximum proliferation rate was found at 3dN and its values were around double those of controls at all stages analyzed (Fig 7C). To better define cell types and the location of cells that were proliferating, BrdU was administered to mice two hours prior to sacrificing them and its incorporation into cells progressing through the cell cycle was assessed by immuno-localization. In the case of *Nkx2-1-Cre; Igf1r*^*fl/fl*^ mice, sections were co-stained with Scgb1a1 and analyzed by confocal microscopy. This allowed us to quantify proliferating epithelial cells in terminal airways (BrdU^+^), and identify cycling club cells among them (BrdU^+^/Scgb1a1^+^) during epithelial regeneration after the naphthalene injury. Since we also noticed an increased presence of BrdU^+^ cells under the epithelial basement membrane, immersed in the smooth muscle layer of the airway, these were also quantified. Results are shown in S8 Fig, with representative details of 3dN and 7dN stages in Fig 7D–7G and their quantification in Fig 7H. As expected, after naphthalene treatment the number of BrdU^+^ cells was increased in all groups of both genotypes at 3dN, but without differences between genotypes. At 7dN, the proportion of total BrdU^+^ epithelial cells and BrdU^+^ cells under the basement membrane was significantly decreased in control mice with respect to 3dN, whereas the *Nkx2-1-Cre; Igf1r*^*fl/fl*^ mutants still maintained high levels. The number of total BrdU^+^ and BrdU^+^/Scgb1a1^+^ bronchiolar epithelial cells were also significantly increased in the mutants with respect to the controls. At 14dN, the means of BrdU^+^ cells were significantly decreased in the three groups of control mice when compared to 3dN, but not in the*Nkx2-1-Cre; Igf1r*^*fl/fl*^ mutants. At this stage, total BrdU^+^ cells in the bronchiolar epithelium of mutant lungs were still significantly increased with respect to the controls. Although not quantified, BrdU immunostaining data obtained from lungs of *Scgb1a1-Cre; Igf1r*^*fl/fl*^ mice also reflected a trend toward this phenotype (S4 Fig, panels I-P). Data obtained with BrdU staining is in accordance with the results obtained with Ki67 staining, and altogether they reinforce the result that bronchiolar epithelial increased proliferation in Igf1r epithelial-specific deficient mice is due to altered and/or delayed differentiation of bronchiolar epithelial cells, mainly affecting club cells.

To explain the increased cell proliferation rates found in the airway epithelium of *Nkx2-1-Cre; Igf1r*^*fl/fl*^ terminal bronchioles, despite their reduced cell density, cell death was determined in lungs of NT and 7dN mice using TUNEL (TdT-mediated dUTP nick labeling immuno-staining). However, we did not find differences in apoptotic rates between genotypes (data not shown). Actually, apoptotic rates in the entire lung were low, and it is difficult to assume that apoptosis is physiologically relevant in this context. Accordingly, similar results were previously described in the lung and liver of *Igf1r* postnatal conditional mutants [31]. Considering that alveolar macrophages could be involved in removing cells or cellular debris by efferocytosis in the lungs of *Nkx2-1-Cre; Igf1r*^*fl/fl*^ mutant mice, the presence of these macrophages was also evaluated by immunostaining using the F4/80 macrophage marker in NT and 7dN lungs of both genotypes. Again, their quantification did not reveal any differences, either in the surroundings of the terminal airways or in the alveolar parenchyma (S9 Fig).

Given that Igf1r signaling was reported to induce cellular senescence in bronchiolar epithelial cells [47], and taking the advantage of the fact that naphthalene treatment induces bronchiolar epithelium to senesce [48], we checked senescence levels in the airway epithelium of the *Igf1r* mutants. To do so, immuno-staining for the DNA damage response and p21 senescence marker [48] was performed in bronchiolar epithelium of NT and 14dN of *Nkx2-1-Cre; Igf1rfl/fl* mice. We did not find significant differences in numbers of p21^+^ cells, either between genotypes at any stage, or between stages in any genotype, although there was a trend toward more senescent cells at 14dN in both genotypes (data not shown).

### Altered expression in epithelial differentiation markers, IGF genes and Igf1r signaling mediators in *Nkx2-1-Cre; Igf1r*^*fl/fl*^ lungs during regeneration after naphthalene injury

**Fig 8:**
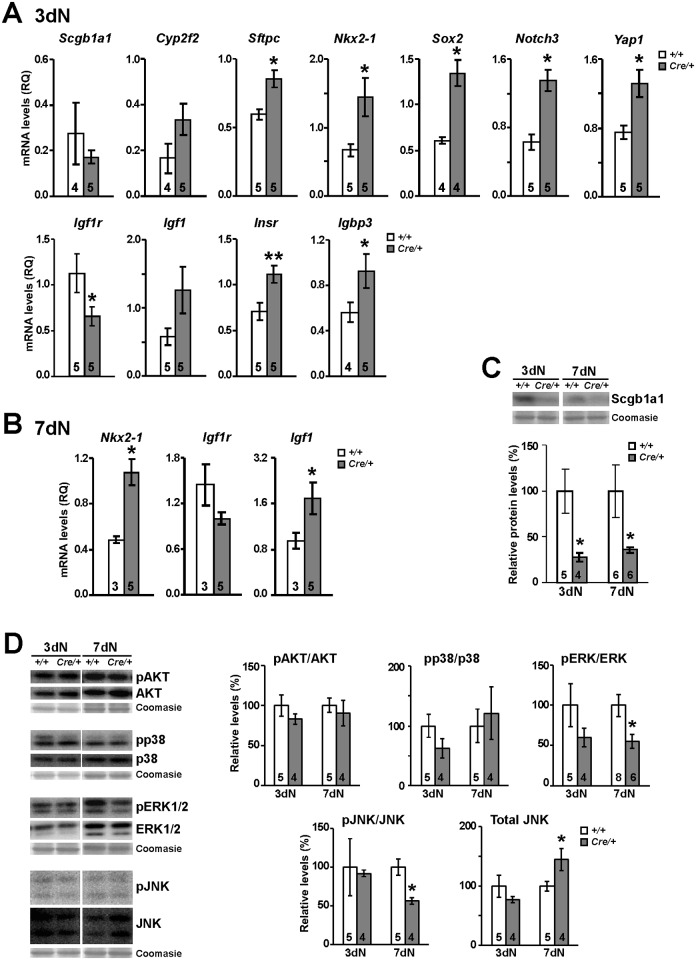
mRNA expression and signaling mediator levels of epithelial cell markers and IGF system genes in lungs of *Nkx2-1-Cre; Igf1r*^*fl/fl*^ mutant mice during regeneration after naphthalene injury. **(A)** mRNA expression levels of IGF system (*Igf1r*, *Igf1*, *Insr* and *Igfb3*), bronchiolar epithelium markers (*Scgb1a1*, *Cyp2f2* and *Sftpc*) and differentiation regulators (*Nkx2-1*, *Sox2*, *Notch3* and *Yap1*) in 3dN total lung extracts. Note that the reduced mRNA levels in *Igf1r* generate increased mRNA in *Insr*, *Igfbp3* and in epithelial precursor-related genes. **(B)** mRNA expression levels of *Igf1r*, and *Igf1* and *Nkx2-1*, in 7dN lungs. *Igf1* and *Nkx2-1* were the unique genes found with significant changes at this stage. **(C-D)** Representative Western blots and their graphical representations after quantification by densitometry for expression of Scgb1a1 (C) and phosphorylation and total levels of IGF signaling mediators (D), including phosphor-(p)-AKT and total Akt, pp38 and total p38, pERK1/2 and total ERK1/2, as well as pJNK/SAPK and total JNK/SAPK, using total lung extracts at 3dN and 7dN stages. Graphs represent blot band densitometric measurements after total protein loading normalization, using either Coomasie staining, or total content of each protein. Note the decreased levels of pERK and the increased levels of total JNK in mutant lungs at 7dN. Numbers in bars indicate number of mice analyzed. *+/+*, *Igf1r*^*fl/fl*^ and *Cre*/+, *Nkx2-1-Cre; Igf1r*^*fl/fl*^genotypes. Values in graphs show mean ± SEM. *, p<0.05; **, p<0.01.

To determine if the lack of Igf1r in lung epithelial cells during repair after naphthalene challenge alters the expression of bronchiolar epithelial cell type-specific markers and epithelial differentiation markers at the transcriptional level, we analyzed the expression of *Scgb1a1*, *Cyp2f2*, *Sftpc*, *Nkx2-1*, *Sox2*, *Notch3* and *Yap1* by qRT-PCR at 3dN and 7dN stages in total mRNA obtained from lungs of *Igf1r*^*fl/fl*^ and *Nkx2-1-Cre; Igf1r*^*fl/fl*^ mice. We found significantly increased expression in *Sftpc*, *Nkx2-1*, *Sox2*, *Notch3* and *Yap1* genes at 3dN (Fig 8A, upper panel), and increased levels of *Nkx2*.*1* at 7dN (Fig 8B and data not shown). Following the same criteria and looking for possible compensatory effects on the expression of IGF system genes, we determined the levels *Igf1*, *Insr* and *Igfbp3*, in addition to *Igf1r*. *Igf1r* mRNA levels were found to be significantly reduced at 3dN, but not at 7dN (Fig 8A and 8B), and conversely, *Igf1* mRNA levels were found to be significantly increased only at 7dN. Finally, it is interesting to note that depletion of *Igf1r* conveyed increased expression of both *Insr* and *Igfbp3* at 3dN (Fig 8A), although differences were not noticed at 7dN (data not shown). Based on the fainter immunostaining in club cells and their reduced numbers at 7dN in the airway epithelium of *Igf1r* mutants, we further tested for changes in Scgb1a1 protein levels using immunoblotting. Analyses at 3dN and 7dN revealed a significant decrease of these protein levels at both stages (Fig 8C). This result further supports a regeneration and differentiation delay of club cells at early stages after their ablation by naphthalene treatment in lungs of *Nkx2-1-Cre; Igf1r*^*fl/fl*^ mutant mice.

Additionally, we compared total protein content and activation levels by phosphorylation of different IGF signaling mediators by Western blot analysis of proteins obtained from lungs of both genotypes at 3dN and 7dN. A downstream canonical mediator for Igf1r signaling in lung development includes the PI3K/Akt pathway [49], however we did not observe differences in AKT expression or activation levels (Fig 8D). Analysis of total protein and phosphorylation levels of p38, ERK1/2 and JNK MAP kinases in total lung protein extracts did not reveal changes in levels of expression or activation in p38 (Fig 8D). However, decreased mean phosphorylation levels of ERK were found at both time points analyzed, but only with statistically significant differences at 7dN (Fig 8D). In addition, a significant reduction in the pJNK/JNK ratio was noticed at 7dN; the differences were not due to phosphorylation levels, but to an increase in total JNK protein levels (Fig 8D).

Altogether, these results suggest that Igf1r deficiency in lung epithelium generates increased expression in epithelial cell precursor-specific genes, induces partial compensatory effects on expression of IGF system genes, including *Insr*, *Igfbp3* and *Igf1* mRNA levels, and also modifies the expression and activation of components of the MAP kinase signaling pathway, leading to decreased activity of ERKs and increased expression of JNK.

## Discussion

The principal aims of this report were to investigate the expression pattern of the insulin-like growth factor type 1 receptor, Igf1r, in the adult mouse lung and its role in regulating the process of airway epithelial regeneration following club cell-specific injury. Analysis of the Igf1r mRNA expression profileduring lung ontogeny revealed a continuous expression in the adult, and immunostaining revealed high levels in the lung epithelium. Conditional deletion of *Igf1r* in the pulmonary epithelium, followed by selective club cell ablation, altered the bronchiolar epithelium homeostasis, causing increased epithelialproliferation and delayed differentiation of club and ciliated cells. These results support a role of Igf1r in maintainingcontrol of bronchiolar epithelial regeneration after injuryin the mouse, and open a possible functional implication of Igf1r in human lung pathological conditions.

Relevance of the functional implication of IGF system genes in the lung is supported by their almost constitutive expression during mouse lung ontogeny. Among all the different IGF/Insulin genes, only expression of *Igf2*and *Igfbp1* were found absent in the adult lung. Accordingly, down-regulation of *Igf2* expression in postnatal mouse lung was previously described[34], extra-pancreatic insulin-production was reported in multiple organs, but not in the lung[50], and the Igfbp1 primary source was reported to bethe liver [51]. Absence of *Igf2* expression in the adult lung also reinforces the role of Igf1 as the major Igf1r ligand with auto/paracrine actions in this tissue, as demonstrated during fetal pulmonary development [29]. Actually, adult mouse lungs displayed the highest level of Igf1r activation of any organ in the mouse upon challenge with Igf1 [52]. This local auto/paracrine action of Igf1/Igf1rcould be relevant not only in airway epithelial cellregeneration, but also foralveolar macrophagefunction, a cell type that also expresshigh levels of both proteins[53, 54]. Furthermore, Igf1r expression in smooth muscle and endothelial cells of pulmonary blood vessels also indicates a role of this receptor in lung vasculature, as described elsewhere [55, 56].

*Igf1r* deletion and reduction of Igf1r expression was obtained in adult micein both mutant lines, *Nkx2-1-Cre; Igf1r*^*fl/fl*^and *Scgb1a1-Cre; Igf1r*^*fl/fl*^, either in the entire lung epithelium or limited to airway club cells, respectively. This was as expected based on previously reported Cre activity for *Nkx2-1-Cre* and *Scgb1a1-Cre* transgenes [40, 41, 57]. It is relevant to mention that Scgb1a1-Cre; Igf1rfl/fl mice showed a very low and variable efficiency in Igf1r depletion in club cells, even in the epithelium of terminal bronchioles. Thus, the lack of Igf1r staining in club cells varied in different lung areas of the same mutant and between animals; some mutant mice did not even lack Igf1r in any of these cells, indicating a low efficiency of the Scgb1a1-Cre transgene in deleting Igf1r gene floxed sequences. On the other hand, despite the fact that *Nkx2-1-Cre; Igf1r*^*fl/fl*^ mutants showed Igf1r depletion inAEC2 cells and that Igf1r signaling was previously reported to be involved in differentiation of these cells [19, 58], adult mice did not show the expected phenotype in the lung alveolar compartment. Furthermore, although Cre expression in lung epithelium of *Nkx2-1-Cre*transgenic mice was described from early lung organogenesis [40, 41], and lack of Igf1/Igf1r signaling revealed a delay and/or altered cell differentiation during embryonic development [26, 28, 29, 59], bronchiolar epithelial alterations in *Nkx2-1-Cre; Igf1r*^*fl/fl*^mutants were not observeduntil mouse adulthood. These results could indicate that the lack of Igf1r in lung epithelium neither significantly alters proper lung development until mouse adulthood nor is required for AEC2 differentiation. In addition, absence of the phenotype could be due to low and/or variable efficiency of floxed sequencesin the *Igf1r* locus by Cre recombinase depending on the cell types and mouse developmental stage. Accordingly, inconstant lung epithelial phenotypes were common in both mutant lines, and different degrees of Cre efficiency, depending on tissues and cell types, were previously reported in a different *Igf1r*^*fl/fl*^ conditional mutant mouse [31].

Since phenotypic changes in lungs of these *Igf1r* mutant mice are noticed even before the naphthalene treatment, and considering that some of these histopathological changes in the bronchiolar epithelium, including lower height and cell density, elongated nuclei, interruptions in epithelial continuity and increased proliferation rates, were recently reported by our group in a different mutant mouse line with a generalized postnatal conditional deletion of *Igf1r*[31], a role of Igf1r in airway epithelial homeostasis is definitively demonstrated. Interestingly, among the different airway cell types, club cells emerge as the most dependent on Igf1r. The fact that club cells in terminal bronchioles are highly abundant [2], and that they express prominent levels of Igf1r (this report) would explain why epithelial club cells in terminal bronchioles show the most conspicuous phenotype when they lack this protein. In agreement, airways of *Scgb1a1* knockout mice also show some of the histopathological features observed in the Igf1r mutants, including the flattened appearance and hyperproliferation of bronchiolar epithelial cells [3].

Analysis of the recovery of the bronchiolar epithelium after treatment with naphthalene in *Igf1r* mutants further demonstrates the relevant role of Igf1r in regulating airway epithelial repair kinetics. Actually, in normal mice expression of *Igf1r* is highly induced during epithelium recovery, and to a lesser extent *Igf1* and *Insr*, and the *Igf1* ligand is prompted earlier than the receptors. The absence of *Igf1r* appeared to haveno observable effect on the extent of initial club cell injury, and this is a reasonable assumptionsince Igf1r is not known to regulate the expressionof genes involved in protectionagainst the cytotoxicity of xenobiotics. However,during epithelial recovery *Igf1r* deficiency caused increased cell proliferation rates, more prominent at early stages,and delayed differentiationafterwards, first noticed in club cells and later on also affecting ciliated cells. This is acceptablegiving that in airways epithelialcell hierarchy club cells are considered to havefirst originated from basal cell precursor and subsequently they are able to self-renew and give rise to both ciliated and globet cells [1, 60]. It is worth mentioning that Igf1r signaling was previously involved in cilia formation in association with cell proliferation and differentiation [61–63].

After systemic treatment of mice with naphthalene, club cells that express cytochrome Cyp2f2 die, remaining ciliated cells spread to cover the denuded matrix and the epithelium is restored by proliferation of naphthalene-resistant club cells adjacent to NEBs and in BADJs[9, 13, 64]. These cells characterized by co-expression of Sftpc and Scgb1a1 at low levels were proposed as putative bronchioalveolar stem cells(BASCs) [65]. BASCs proliferation and differentiation into mature airway epitheliuminvolve the induction of regulatory genes such us *Sox2*, *Notch3*, *Yap1* and *Nkx2-1*[46, 64,66–70]. Consequently, increased mRNA levels of these genes, including *Sftpc*, found at 3dN in the lungs of *Nkx2-1-Cre; Igf1r*^*fl/fl*^ mutant mice,would support the undifferentiated status andthe highercell proliferation rates reported in their airway epithelial linings after injury. Accordingly, the highest significant increase in bronchiolar epithelium proliferation rates was found at 3dN in the mutant lungs. These results sustain the notion that lack of Igf1r in the pulmonary epithelium augments the appearance of epithelial progenitors and increases the proportion of undifferentiated airway epithelial cells by increasing proliferation and halting differentiation at early stages of regeneration after injury.

Paradoxically, despite the fact that Igf1r has a predominant role as an inducer of cell proliferation and survival (reviewed in [16, 71]), both *Igf1r* mutant mouselines showed increased cell proliferation rates in epithelial cells of distal airways. Accordingly, lack of Igf1 and Igf1r was reported to promote cell proliferation and/or to alter epithelial differentiation not only in the lung [25, 26, 28, 29, 31], but also in the prostate, thyroid gland, liver and nervous system[31, 44, 72, 73]. Furthermore, limited activity of Igf1r accelerates tumorigenesis and promotes more aggressive phenotypes in prostate and breast cancer in mice [72, 74]. Despite this hyperproliferative effect we did not see any hyperplasias in the airway epithelium of the *Igf1r* conditional mutants. It could be of interest if they were susceptible after being challenged with a protumoral driver signal. Notwithstanding the prominent epithelial cell proliferation found in the *Igf1r* conditional mouse mutants, apoptotic rates were unaltered. In accordance, hypoplasic prenatal lungs of IGF1-deficient mice and lungs of *UBC-CreERT2; Igf1r*^*fl/fl*^ adult mutant mice with a generalized induced deletion of Igf1r have also been reported to be hyperproliferative without changes in apoptosis [29, 31]. In any case, understanding the kinetics of cell death in each model system is critical, and proper timing of the experimental design may be crucial to identify apoptosis [75]. An excessive generation of incorrectly differentiated airway epithelial cells in I*gf1r* mutants could also preclude increased cell clearing by engulfment eitherby alveolar macrophages or neighboring epithelial cells as described elsewhere [76]. Nevertheless, we did not find any evidence of them, either in changes in macrophages numbers or in morphological sing of epithelial phagocytic activity.

*Igf1* and *Ifbp3* mRNA levels were found to be increased in the lungs of *Nkx2-1-Cre; Igf1r*^*fl/fl*^ mutant mice at early stages after the naphthalene challenge, probably as a consequence of a compensation mechanism among different Igf/Insulin system genes, as previously reported[29]. Furthermore, Igf1 and Igfbp3 systemic levels rise in mice with a pharmacological blockade of Igf1r, and also in patients with mutations of this gene [23, 52, 77–79]. Since Igf1 and Igfbp3 levels have been involved in controlling colonic epithelial stem cell function [80], upregulation of their expression in the lungs of our *Igf1r* mutants could alter BASC function during airway epithelial regeneration. Additionally, Igfbp3 overexpression could be the consequence of a protective molecular mechanism in response to lung damage or pathological conditions, as demonstrated respectively in mice and patients [21, 81–84]. Expression of *Insr* in the rodent lung was verified some time ago, at both fetal and adult stages [85, 86], and alterations in insulin signaling, including diabetes and metabolic syndrome have been associated with an increased risk in lung diseases (reviewed in [87]). Lungs of *Nkx2-1-Cre; Igf1r*^*fl/fl*^ mice also show upregulation of *Insr* mRNA after the naphthalene challenge. Accordingly, Insr signalingwas found to improve when Igf1r availability was compromisedin endothelial cells [55, 56]. Furthermore, upregulation of *Insr* in lungs of *Igf1r*-defient mice could contribute to hyperproliferation of airway epithelium as described in tumors. Thus, INSR functionally enhances multistage tumor progression and conveys intrinsic resistance to IGF1R targeted therapyin pancreatic islet tumor growth[88]. Moreover, IGF signaling has been previously involved in keeping human lung cancer cell stemness while IGF1R downregulation, in conjunction with INSR inhibition, was more effective in blocking IGF- and insulin-mediated signaling and growth in cancer cells compared to single-receptor targeting alone [89, 90].

Akt and ERK, p38 and JNK MAP kinases are well known as canonical Igf1r signaling mediators, and they were previously implicated in controlling embryonic lung development as well as adult BASC homeostasis [91–95]. The finding of a significant decrease of ERK activation and an increase in total JNK expression at 7dN, coinciding with the peak of club cell differentiation,would indicate that club cell regeneration relays an Igf1r appropriate control of the Akt and MAP kinase pathway. In this sense, it was recently reported that Igf1r signaling contributes to bronchiolar epithelium cell senescence by activation of Akt/mTOR [47, 96], and that naphthalene exposure,combined with BrdU injection,also induces bronchiolar epithelial cells to senescence and inflammation by regulating MAPK activity[48]. Yet,Igf1r depletion in airway epithelial cells of *Nkx2-1-Cre; Igf1r*^*fl/fl*^ mutants did not show evidence of cell senescence protection. The reason could be compensatory mechanisms, dilution of airway epithelium effects on total lung extracts, or an inadequate window timing selection for the analysis.

In summary, the findings obtained from this study contribute to the field of IGF biology by revealing their possible novel role in the control of airway epithelial cell differentiation. It is important to compare the high similarities in both, mRNA total expression levels of IGF system genes and the Igf1r protein expression pattern at the cellular level described here in the adult mouse lung, with those recently reported in humans[37]. In addition, data reported in this study clearly show that Igf1r plays an important role in regulating bronchiolar airway epithelial repair kinetics following club cell-specific ablation by keeping an adequate balance between proliferation and differentiation in basal progenitor cells after injury. These findings potentially indicate a possible involvement of Igf1r in regulating gene expression in the context of repair occurring in response toairway epithelial injury in thesetting of various pulmonary diseases, such as asthma andchronic obstructive pulmonary disease. Identification of early events that contribute to the establishment of chronic lung disease has been complicated by the variable involvement of the airway compartment in the complex physiology of end-stage disease. Therefore, a better understanding of theunderlying mechanisms by which IGFs promote self-renewal and differentiationof lung cells will be crucial inidentifyingnew therapeutic approachesfor lung diseases. Furtherstudies by development of animal models of lung diseases that combine pulmonary injuries, e.g. resembling asthma or COPD, withaltered expression of IGF genes targetingspecific cell typesare required in order to elucidate the exact role of Igf1r and other IGF genes in pulmonarypathological conditions.

## Materials and Methods

### Ethics Statement

All experiments and animal procedures were carried out in accordance with the guidelines laid down by the European Communities Council Directive of 24 November 1986 (86/609/EEC) and were revised and approved by the CIBIR Bioethics Committee (refs. 12/11 and 7/12) (Logroño).

### Generation of Nkx2-1-Cre; Igf1r^fl/fl^ and Scgb1a1-Cre; Igf1r^fl/fl^ mice

*Nkx2-1-Cre; Igf1r*^*fl/fl*^ and *Scgb1a1-Cre; Igf1r*^*fl/fl*^ double transgenic mice were created in two generations by mating hemizygous *Nkx2-1-Cre(Tg(Nkx2-1-cre)2Sand*; MGI:3773076) [97], and *Scgb1a1-Cre (Tg(Scgb1a1-cre)1Tauc*; MGI:3610310) [42] transgenics, with homozygous *Igf1r*^*fl/fl*^mutants (*Igf1*^*rtm1Jcbr*^; MGI:3818453) [98]. All three lines were in an enriched C57BL/6 genetic background. *Nkx2-1-Cre; Igf1r*^*fl/+*^ and *Scgb1a1-Cre; Igf1r*^*fl/+*^ heterozygous mice generated in F1 were backcrossed with *Igf1r*^*fl/fl*^ to yield an F2 with equal proportions of four genotypes, among them both double transgenic mice with the genotypes of interest, *Nkx2-1-Cre; Igf1r*^*fl/fl*^and *Scgb1a1-Cre; Igf1r*^*fl/fl*^, and *Igf1r*^*fl/fl*^control mice. Finally, for experimental purposes *Nkx2-1-Cre; Igf1r*^*fl/fl*^ and *Scgb1a1-Cre; Igf1r*^*fl/fl*^double transgenic mice were crossed with *Igf1r*^*fl/fl*^to directly generate descendants with equal proportions of both parental genotypes.

### Mouse genotyping

DNA from mouse tails and tissues was obtained as previously described [31], and genotyped by standard PCR analysis using specific primers for each transgene designed as shown in S1 Fig, panels A-D. Presence of *Nkx2-1-Cre* transgene was detected using primers P1 (5′- CCACAGGCACCCCACAAAAATG-3′) and P2 (5′-GCCTGGCGATCCCTGAACAT -3′) [97], in combination with two additional primers for the *IL2* gene, IL2F (5′- CTAGGCCACAGAATTGAAAGATCT-3′) and IL2R (5’-GTAGGTGGAAATTCTAGCATCATCC-3’), used as an internal PCR positive control. After amplification by PCR (94°C for 3 min; 30 cycles of 94°C for 30 s, 56°C for 30 s, and 72°C for 1 min; and finally 72°C for 7 min), they rendered 666 and 325 bp-long amplicons, respectively. *Scgb1a1* transgene was identified with primers for a generic Cre gene identification, F (5′- GCGGTCTGGCAGTAAAAACTATC-3′) and R (5′- GTGAAACAGCATTGCTGTCACTT-3′), in combination with the above-mentioned *IL2* primers. PCR (94°C for 5 min; 30 cycles of 94°C for 30 s, 60°C for 30 s, and 72°C for 30 s; and 72°C for 7 min.) rendered 100 and 325 bp-long amplicons, respectively. *Igf1r* wild type (*wt*) or flox (*fl*) alleles, and *Igf1r* deletion, were determined as described [31] (S1 Fig).

### Naphthalene treatment

Naphthalene (Sigma, St Louis, Mo) was dissolved in corn oil (Sigma) at 25 mg/ml and administered to *Nkx2-1-Cre; Igf1r*^*fl/fl*^ and *Scgb1a1-Cre; Igf1r*^*fl/fl*^double transgenics and to the control *Igf1r*^*fl/fl*^mice intraperitoneally (250 mg/kg) with a single dose at 12–14 weeks of age as described elsewhere [8]. Vehicle (corn oil) was tested as a control. Animals were monitored for adverse effects, and if these become apparent, treatment was stopped. Groups of 3–9 mice per genotype were killed at different stages of recovery, including three days after naphthalene treatment (3dN), 7dN, 14dN, and 24dN.

### BrdU administration, lung dissection, histology, immunostaining, TUNEL and Western immunoblotting

Mice were given intraperitoneal injections of 10 μl BrdU (Roche, Basel, Switzerland) per gram of body weight two hours before sacrifice. Mice were killed by intraperitoneal injection of (300 mg/kg ketamine, 30 mg/kg Xylazine in saline). Following lung dissection, right lobes (superior, middle, inferior and post-caval) were separated and snap frozen in liquid nitrogen to be used for DNA (post-caval) extraction, RNA analysis (inferior) and protein immunoblotting (superior). Left lung lobes were inflated through the left bronchus with neutral buffered formalin (NBF) and help of a syringe with an attached needle, inmersion-fixed in NBF for 8–14 hours, dehydrated through graded ethanols, and embedded in paraffin following standard methods. Details on histological, immunostaining, TUNEL detection of apoptotic cells and Western immunoblotting analysis are available in S1 Appendix(Supporting Methods). Primary and secondary antibodies used in immunodetection techniques are listed in S1 Table.

### Morphometric analysis of bronchiolar epithelium

Bronchiolar cellular density and bronchiolar cell height were assessed using 10–21 fields per section of 1 slide from each animal in 400 X fields (7.5 x 10^4^ μm^2^) in a light microscope (Nikon Instruments Inc.), with help of Fiji Open Source image processing software package (http://fiji.sc). To calculate the bronchiolar cell height, 3 representative average cell size measurements (μm) were made on each field. Bronchiolar density was determined by counting hematoxylin-stained nuclei per length unit (mm), and bronchiolar interruptions were counted as the number of gaps per length unit (mm) evaluating 8–48 fields per section on each animal, taking as reference the basement.

### RNA isolation, reverse transcription, quantitative real-time PCR and RNA-seq

Total RNA was obtained from homogenized inferior lung lobes using Trizol ReagentH (Invitrogen, Carlsbad, CA), treated with 2.72 kU/μL RNase-free DNase (Qiagen, Hilden, Germany) and purified through RNeasy columns (Qiagen) following manufacturer instructions. The quantity and quality of total RNA was assessed on a NanoDrop Spectophotometer and an Agilent 2100 Bioanalyzer, respectively. cDNA was generated using SuperScript II First-Strand Synthesis System (Invitrogen, Carlsbad, CA) according to manufacturer guidelines. Triplicates of cDNA samples were amplified by qRT-PCR on a 7300 Real Time PCR instrument (Applied Biosystems, Foster City, CA) using SYBR green master mix (Applied Biosystems). 18S ribosomal RNA was used as endogenous control to normalize results. Information on primers used in qRT-PCR is shown in S2 Table. Details on RNA-seq analysis are included in S1 Appendix(Supporting Methods).

### Quantification of Scgb1a1/GluTub, Macrophages (F4/80) and p21 positive cells

Quantification of Scgb1a1 and/or GluTub, positive cells in bronchiolar epithelium were made by counting 8–48 fields per animal in 400X fields (15 x 10^4^ μm^2^) using a S5 confocal microscope (Leica Microsystems). The results were expressed as the number of Scgb1a1/GluTub-positive cells per unit length (mm). Similarly, quantification of F4/80 and p21 positive cells in alveolar and bronchiolar areas was made by counting 10 fields per animal of each area, and the results were expressed as the number of macrophages or p21^+^ cells per area (mm^2^).

### Quantification of cell proliferation by BrdU and Ki67 immunostaining

Quantification of BrdU or BrdU/Scgb1a1 positive bronchiolar cells was made by using 17–46 fields per section per animal in 400X fields (15 x 10^4^μm^2^) in a S5 confocal microscope (Leica Microsystems). The final results were expressed as the number of BrdU positive bronchiolar cells per area (mm^2^).

The proportion of Ki67-positive proliferating cells was determined as described [31] (S1 Appendix) (Supporting Methods). Ratios of Ki67^+^ cells to total cell numbers in 400X fields (7.5 x 10^4^ μm^2^) were determined using a light microscope (Nikon Instruments Inc.). Cell counting for Ki67^+^ cells was carried out using 6–30 fields of terminal bronchioles areas, and 10 fields in alveolar areas, using 1–2 slides from each mouse.

### Quantification of Igf1r immunofluorescent staining

To quantify Igf1r total fluorescence in pulmonary bronchiolar and alveolar type 2 cells (Fig 2), an outline was drawn around each cell, and area and mean fluorescence were measured along with several background readings performed with Fiji Open Source image processing software package. The total corrected cellular fluorescence (TCCF) = Integrated density–(area of selected cell x mean fluorescence of background readings) was calculated as previously described [99]. In both cell types, Igf1r relative fluorescence was measured in 10 cells from 4 different lung fields and using 4 animals per genotype.

### Statistical analysis

All measurement data are expressed as mean ± SEM. For statistical analysis of the data, the SPSS^®^ data mining software (version 19) was used. Differences between genotypes were performed using the Mann-Whitney U test. Differences among conditions in the same group were analyzed using non-parametric tests, either Wilcoxon for comparison between two conditions in the same group or Kruskall Wallis for comparison among three or more conditions with help of appropriated post-hoc tests. *p* < 0.05 was considered statistically significant.

## Supporting Information

S1 Appendix Supporting Methods. (DOCX)

S1 Fig *Nkx2-1-Cre* and *Scgb1a1-Cre* transgenes, *Igf1r*floxed locus organization, and PCR strategy for both mouse genotyping and *Igf1r*deletion identification. **(A-B)***Nkx2-1-Cre* (A) and *Scgb1a1-Cre* (B) transgene elements and location of their respective primers (P1/P2 and F/R) for PCR genotyping. **(C)** Genomic DNA organization in alternative allelic forms of the *Igf1r* locus (*wt*, *floxed* and *deleted*), and specific primers (F1, F3 and R1) used for *Igf1r* locus analysis by PCR. (**D)** Expected amplicon sizes in PCR assays to identify the presence of *Scgb1a1-Cre* or *Nkx2-1-Cre* transgenes and the different *Igf1r* allelic forms. IL2 primers were used as constitutive positive controls when genotyping hemizygous *Nkx2-1-Cre* or *Scgb1a1-Cre* mice. **(E)** PCR mouse genotyping in tail DNA to identify *Igf1r* locus alleles (*wt* or *floxed*), and the presence of *Cre* transgenes using P1/P2 (for *Nkx2-1-Cre*) and F/R (for *Scgb1a1-Cre*), in combination with *IL2* primers as an internal control. **(F)** PCR assays to determine the *deleted* allele of *Igf1r* (Δ) using F3/R1 primers on genomic DNA obtained from lung and tail of *Igf1r*^*fl/fl*^*(+/+/fl/fl)* as control mice, and *Scgb1a1-Cre; Igf1r*^*fl/fl*^*(Cre/+/fl/fl)* as mutant mice. The 491 bp fragment of the *deleted* form *(Δ*) is present only in lungs of *Cre/+/fl/fl* animals. **(G)** PCR assays of genomic DNA obtained from different tissues of *Scgb1a1-Cre; Igf1r*^*fl/fl*^. Note the presence of the deleted allele (Δ) in the tracheal epithelium (TrEp), and in the proximal (PrLu) and distal lung (DiLu) but not in the liver (Li), spleen (Sp), kidney (Ki), or testis (Te). bp, base pairs.
(PNG)

S2 Fig Reduced Igf1r expression in terminal bronchioles of adult *Scgb1a1-Cre; Igf1r*^*fl/fl*^ double transgenic mice. **(A-D)** Images of immuno-staining for Igf1r (green labeling in left panels) counter-stained with Scgb1a1 (red labeling in central panels) to identify club cells in terminal bronchioles, in lungs of *Igf1r*^*fl/fl*^ (A, C) and *Scgb1a1-Cre; Igf1r*^*fl/fl*^ (B, D) obtained from six months (A, B) and one year (C, D) old mice. Right panels are merged images of Igf1r/green (left panels) and Scgb1a1/red (central panels) to show co-localization of both markers in the club cells (orange), in addition to nuclear DAPI staining. Note that in control mice, Igf1r (green arrows, left panels in A and C) co-stained abundant Scgb1a1^+^ club cells (orange arrows, right panels in A and C). However, distal bronchiolar epithelium of *Scgb1a1-Cre; Igf1r*^*fl/fl*^ mice show a strong reduction in the number of Igf1r^+^ (green arrows, left panels in B and D), sometimes organized in epithelial areas with complete lack of Igf1r expression (Δ). Lack of Igf1r correlated with a reduction in number and size of Scgb1a1^+^ club cells (central and right panels in B and D), and many of the remaining Scgb1a1^+^ epithelial cells, did not express Igf1r (colored in red, right panels in B and D). al, alveolus; tb, terminal bronchiole. Scale bar in D (left panel): 50 μm; applies to all panels.
(PNG)

S3 Fig Histological analysis of terminal bronchioles in *Nkx2-1-Cre; Igf1r*^*fl/fl*^mutant mice during repair after the naphthalene challenge. **(A-D)**Representative H&E staining of lung terminal bronchioles sections obtained from control *Igf1r*^*fl/fl*^ (A, C) and mutant *Nkx2-1-Cre; Igf1r*^*fl/fl*^(B, D) three months old non-treated mice (NT) mice, at low (A-B) and high magnification (C-D). Note that the terminal bronchiolar epithelium of mutant mice shows epithelial flattening (red segment), thinner club cells, with absence of their *cupulated* shape (present in controls; red arrows in C), presence of aberrant ellipsoid nuclei (green arrows) (D) and interruptions in epithelial continuity (black arrowheads in F, G and J). **(E-L)**H&E staining of terminal bronchioles in control and mutant mice after naphthalene treatment at three (3dN)(E-F), seven (7dN)(G-H), fourteen (14dN)(I-J) and 24 (24dN)(K-L) days of recovery after challenge. Conditional mutant lungs show epithelial cells with ellipsoid nuclei protruding in the bronchiolar lumen (green arrows) and lack of club cells compared with the controls. Those observations are more evident at 7dN and 14dN where there are extensive areas with lack of “cupulated” club cells (Δ, red line). See morphological quantifications in Figs 3E and 5G–5E. al, alveolus; NT, no treatment; tb, terminal bronchiole. Scale bar in L: 50 μm in A-B, E-L. Scale bar in D: 10 μm in C-D.
(PNG)

S4 Fig Histopathological and proliferation analysis in bronchioles of *Scgb1a1-Cre; Igf1r* ^*fl/fl*^ mice after the naphthalene injury. H&E histological (A-H) and BrdU immuno-histochemical (I-P) stainings to respectively evaluate the histology and proliferation in three months old control (*Igf1r*^*fl/fl*^) and mutant (*Scgb1a1-Cre; Igf1r*^*fl/fl*^*)* mice, either before (NT)(A-B; I-J) or after the naphthalene treatment at different stages of recovery: three (3dN)(C-D; K-L), seven (7dN)(E-F; M-N) and fourteen (14dN)(G-H; O-P) days. Note that the bronchiolar epithelium in mutant mice do not show evident histological alterations in club cells (B), compared with controls (A) (red arrows point to normal club cells). In terminal bronchioles of naphthalene treated mice, the *Scgb1a1-Cre; Igf1r* ^*fl/fl*^ mutant lungs show more club cells with altered morphology (green arrows) and less proportion of club cells (red arrows). At 14dN, extensive areas of the epithelium appear lacking protruding cupules of club cells (Δ, green line in H). After immuno-staining for BrdU (administered 2 h label prior sacrifice) the number of BrdU^+^ cells (labeled in brown, black arrows) in NT, 3dN and 14dN mice did not show evident differences between genotypes (I-J, K-L and O-P). However note the increased number of BrdU^+^ labeled at 7dN in the mutants (black arrows in N). al, alveolus; NT, no treatment; tb, terminal bronchiole. Scale bar in H: 20 μm in A-H. In P: 50 μm in I-P.
(PNG)

S5 Fig Delayed regeneration of club and ciliated cells in bronchiolar epithelium of *Nkx2-1-Cre; Igf1r*^*fl/fl*^ mutants after the naphthalene challenge. Representative images of immuno-staining to identify Scgb1a1^+^ cells in red, and ciliated/GluTub^+^ in green in lungs of *Igf1r*^*fl/fl*^ (A, C, E, G, K) and *Nkx2-1-Cre; Igf1r*^*fl/fl*^ (B, D, F, H, L) before (A-B) and after (C-L) naphthalene treatment. Counterstain with DAPI in blue label nuclei. Note the lower proportion of club (red arrows) and ciliated cells (green arrows), and the increased presence non-labeled epithelial cells (blue arrows) in *Nkx2-1-Cre* mutants. These phenotypes were more evident at 7dN and 14dN stages. See quantifications in Fig 6E. al, alveolus; NT, no treatment; tb, terminal bronchiole. Scale bar in L: 50 μm, applies to all panels.
(PNG)

S6 Fig Delayed recovery in Igf1r and Scgb1a1 expression in club cells of *Scgb1a1-Cre; Igf1r*^*fl/fl*^ lungs after the naphthalene injury. Immuno-staining for Igf1r (green labeling in left panels) and Scgb1a1 (red labeling in central panels) obtained from terminal bronchioles of *Igf1r*^*fl/fl*^ (A, C, E, G, I) and *Scgb1a1-Cre; Igf1r*^*fl/fl*^(B, D, F, H, J) mice before (NT) and after different days of the naphthalene treatment (dN). Right panels are merged images of Igf1r/green (left panels), Scgb1a1/red (central panels) to show co-localization (orange) of both markers in club cells, in addition to nuclear DAPI staining. **(A-B)** Terminal bronchioles of NT mice. Note that in control mice, Igf1r (green arrow, left panel in A) co-stained abundant Scgb1a1^+^ club cells (orange arrow, right panel in A). However, distal bronchiolar epithelium of *Scgb1a1-Cre; Igf1r*^*fl/fl*^ mice show scarce of Igf1r^+^/Scgb1a1^+^ cells (green, red and orange arrowheads in B), sometimes organized in epithelial areas with complete lack of Igf1r expression (Δ) (See S2 Fig). **(C-J)**Immuno-staining as described in A-B, after naphthalene treatment at three (3dN)(C-D), seven (7dN)(E-F), fourteen (14dN)(G-H) and twenty four (24dN)(I-J) days after the naphthalene challenge. Note the reduced or complete lack of Igf1r epithelial staining (Δ, white segments in left panels), the delayed regeneration in club cells (reduced proportion of red cells compared to controls, in central panels), and reduced numbers of cells, but still present, that retains Igf1r/Scgb1a1 co-expression (arrowheads). al, alveolus; NT, no treatment; tb, terminal bronchiole. Scale bar in J: 50 μm; applies to all panels.
(PNG)

S7 Fig Increased cell proliferation rates in terminal bronchioles of *Nkx2-1-Cre; Igf1r*^*fl/fl*^ mice at different stages during regeneration after the naphthalene treatment. Representative images of Ki67 immunostaining (brown nuclei and arrows) in terminal bronchioles of *Igf1r*^*fl/fl*^ (A, C, E, G, I) and *Nkx2-1-Cre; Igf1r*^*fl/fl*^ (B, D, F, H, J) lungs before (NT)(A-B) and after three (3dN)(C-D), seven (7dN)(E-F), fourteen (14dN)(G-H) and twenty four (24dN)(I-J) days after the naphthalene treatment. Note the increased presence of Ki67^+^ cells in the epithelium of *Nkx2-1-Cre* mutant mice respect to controls, at all stages. See quantifications in Fig 7C. al, alveolus; NT, no treatment; tb, terminal bronchiole. Scale bar in J: 50 μm; applies to all panels.
(PNG)

S8 Fig Alterations in cell proliferation patterns in terminal bronchiolar epithelium of *Nkx2-1-Cre; Igf1r*^*fl/fl*^ mice during repair after the naphthalene injury. **(A-H)**Immuno-staining for BrdU labeling (green labeled nuclei) in terminal bronchioles of *Igf1r*^*fl/fl*^ (A, C, E, G) and *Nkx2-1-Cre; Igf1r*^*fl/fl*^(B, D, F, H) mice before (A-B) and after three (3dN)(C-D), seven (7dN)(E-F) and fourteen (14dN)(G-H) days after the naphthalene treatment. Sections were co-stained with Scgb1a1 (red labeling) allowing to differentiate three groups of cells: total BrdU^+^ cells in the bronchiolar epithelium (green arrows), BrdU^+^/Scgb1a1^+^ double labeled cells (red arrows) and BrdU^+^ cells under the epithelial basal membrane (blue arrows). Note the increased number of bronchiolar epithelial cells proliferating in the mutants at 7dN (E-F) (green and red arrows), accompanied by an increased number of BrdU labeled cells below the basal membrane nearby the epithelium (blue arrows). See quantification in Fig 7H. al, alveolus; NT, no treatment; tb, terminal bronchiole. Scale bar in H: 50 μm; applies to all panels.
(PNG)

S9 Fig Unaltered numbers of macrophages in alveolar and bronchiolar areas of *Nkx2-1-Cre; Igf1r*^*fl/fl*^ mice during regeneration after the naphthalene injury. The graphic represents the quantification of F4/80 stained cells under the confocal microscope observation in alveolar and bronchiolar areas of control (*+/+*) and mutant (*Cre/+*) mice, in non-treated (NT) and at day seven (7dN) after the naphthalene treatment. Quantification of macrophages in the bronchiolar area corresponds to measurements performed in confocal picture frames of bronchiolar fields counting all F4/80^+^ cells, including those located in the surrounding alveolar parenchyma. Note the reduced presence of macrophages in both genotypes at 7dN, although without significant differences between genotypes.
(PNG)

S1 Table Source and dilution of primary and secondary antibodies used in immunodetection. (DOCX)

S2 Table List of primer sets used in qRT-PCR. (DOCX)
