# Supplementary material for: Involvement of Igf1r in Bronchiolar Epithelial Regeneration: Role during Repair Kinetics after Selective Club Cell Ablation
Source: PLoS One. 2016 Nov 18;11(11):e0166388. doi: 10.1371/journal.pone.0166388 (PMC5115747; doi:10.1371/journal.pone.0166388)
Supplement: S1 Appendix — (DOCX) [file pone.0166388.s001.docx]

***S1. Appendix.* Supporting Methods**

**Histology.** For histological and immuno-histochemical analysis, tissues in paraffin were cut into 3 µm sections, dewaxed and rehydrated by standard methods. Slides stained with hematoxylin and eosin (H&E) were observed and photo-documented using a light microscope (Nikon Instruments Inc.).

**Fluorescent immunostaining.** Paraffin sections (3 µm) were single or double stained for Igf1, Igf1rβ, Nkx2-1, Scgb1a1, Pre-SftpC, Pdpn, Pecam1, F4/80, Cgrp, Glu-Tubulin, and αSMA as elsewhere described (Pais *et al.* 2013. *PLoS One* 8: e83028). With exception of F4/80, antigen retrieval was performed by immersing the slides in a boiling solution of 10 mM Citrate (pH 6.0) for 25 minutes, and followed by a 15 min cool down in the same buffer. For F4/80/Igf1r double staining, slides were immersed in 10 mM TE buffer with Proteinase K (Roche) (20 μg/ml) for 15 min at 37ºC and followed by a 15 min cool down. After antigen retrieval all slides were blocked with 10% serum in PBS-Triton (0.1%) and Glycine (10 mM) for 1 h at room temperature, and incubated at 4ºC overnight with primary antibodies. After washing, sections were incubated for 1 h, with secondary antibodies, and mounted in ProLong Gold Antifade Reagent with DAPI (Molecular Probes) to be examined under a confocal microscope (Leica Microsystems). Origin and dilution of primary and secondary antibodies used are detailed in S1 Table.

**Ki67 immunohistochemistry.** For Ki67 detection a streptavidin-biotinylated immunoperoxidase method was used. Ki67 localization was performed as previously described (López *et al.* 2015. *Transgenic Res* 24: 279-294). Briefly, for antigen retrieval, sections were immersed in a boiling solution of 1 mM EDTA (pH 9.0) for 25 min, and followed by a 15 min cool down in the same buffer. Endogenous peroxide was blocked by 0.2 % H2O2 for 15 min. After blocking with 4 % goat serum in PBS-Triton (0.1 %) and BSA (2 %) for 1 h at room temperature, slides were incubated at 4 ºC overnight with a primary rabbit monoclonal antibody anti Ki67 (Clone SP6, Master Diagnostica) at a 1:200 dilution. Sections treated with biotinylated anti-rabbit antibody (BA-1000, Vector Laboratories), were visualized with avidin-biotin-peroxidase complex (Vector Elite ABC kit; Vector Laboratories) and 3,3-diaminobenzidine substrate (Sigma) under a light microscope (Nikon Instruments Inc.).

**BrdU immunohistochemistry.** For single BrdU detection in Scgb1a1-Cre; Igf1r^fl/fl^ lungs a streptavidin-biotinylated immunoperoxidase method was also followed. For antigen retrieval, sections were immersed in a boiling solution of 1 mM EDTA (pH 9.0) for 25 minutes, and followed by a 15 min cool down in the same buffer. After washed in PBS and immersed in 2N HCl for 5 min at room temperature and 25 min at 37ºC, and in 0.1 M borate buffer (pH 9.5) at room temperature for 5 min, sections were blocked with 10% donkey serum in PBS-Triton (0.1%) and Glycine (10 mM) for 1 h at room temperature and incubated at 4ºC overnight with the primary antibody (S1 Table). Finally, after washing, slides were incubated for 1 h with a secondary antibody and visualized with avidin-biotin-peroxidase as described above. The same protocol for antigen retrieval was followed in case of double immuno-staining for BrdU/Scgb1a1. In this case after incubation with appropriate antibodies, sections were mounted in ProLong Gold Antifade Reagent with DAPI (Molecular Probes) to be examined under a confocal microscope (Leica Microsystems).

**TUNEL analysis.** TUNEL detection of apoptotic cells was performed as described (López et al. 2015). In brief, lung cells undergoing apoptosis were identified using a TUNEL (TdT-mediated dUTP Nick-End Labeling) assay kit (DeadEnd Colorimetric System, Promega) following the manufacturer’s instructions. Apoptotic nuclei stained dark brown were counted in 200X fields using a light microscope (Nikon Instruments Inc.). At least 2 slides (50 μm apart) from each mouse were counted, using three mice per genotype.

**Western immunoblotting.** Total protein was obtained from superior lung lobes homogenized in lysis buffer (New England Biolabs, Frankfurt, Germany) supplemented with protease inhibitor cocktail (Complete Mini, Roche, Mannheim, Germany) using a commercial homogenizer (Precellys 24, Peqlab Biotechnologie GmbH, Erlangen, Germany). Insoluble cell debris was removed by centrifugation (10000 g, 2 min, 4°C) and protein content of the soluble tissue lysate was quantified by absorptiometry at 280 nm against blank lysis buffer. Immunoblotting was performed as described previously ([Reyer](https://correo.riojasalud.es/owa/redir.aspx?C=uQvBg0UV40aiWHbEBVICtcrZ-hMM7NIIP2LN4LERPYdTqyIUI3OnOGMUtvaw_aM-Vo2gCVYz1Qk.&URL=http%3a%2f%2fwww.ncbi.nlm.nih.gov%2fpubmed%2f26105006) *[et al.](https://correo.riojasalud.es/owa/redir.aspx?C=uQvBg0UV40aiWHbEBVICtcrZ-hMM7NIIP2LN4LERPYdTqyIUI3OnOGMUtvaw_aM-Vo2gCVYz1Qk.&URL=http%3a%2f%2fwww.ncbi.nlm.nih.gov%2fpubmed%2f26105006)* [2015](https://correo.riojasalud.es/owa/redir.aspx?C=uQvBg0UV40aiWHbEBVICtcrZ-hMM7NIIP2LN4LERPYdTqyIUI3OnOGMUtvaw_aM-Vo2gCVYz1Qk.&URL=http%3a%2f%2fwww.ncbi.nlm.nih.gov%2fpubmed%2f26105006). *Am J Physiol Endocrinol Metab* 309: E409-E417). In brief, 10 µg of protein were separated by sodium dodecyl sulfate-polyacrylamide gel electrophoresis (SDS-PAGE), transferred to polyvinylidene fluoride (PVDF) membranes and incubated with antiserum after blocking. Detailed information on antibodies is shown in S1 Table. Detection was performed using the Kodak Image Station 4000MM (Stuttgart, Germany) using the ECL Advance Kit (GE Healthcare, Freiburg, Germany). Protein expression or activation was normalized either by the Coomassie Blue Signal (Sawitzky *et al.* 2012 PLoS One 7:e39711) or the total levels of the signaling molecules, respectively.

**RNA-seq.** Deep transcriptome analysis (RNA-seq) was performed in our Institution (Genomics Platform, CIBIR, Logroño, Spain) with help of a next-generation sequencer (Genome Analyzer IIx, Illumina Inc., San Diego, CA), using one microgram of total lung RNA from three-month-old *Igf1r^fl/fl^* normal mice (n=5) (López et al. 2015. *Transgenic Res* 24:279). Briefly, after verifying RNA quality in an Experion Bioanalyzer (BioRad), TruSeq total RNA libraries were generated and sequenced according to manufacturer instructions (Illumina). The 5 libraries were ultrasequenced in one channel of the flow cell using one run of single-end reads with 150 cycles. *Mus musculus* GRCm38.71 (FASTA) from the Ensemble database was used as the reference genome. After removing adapter sequences with the Cutadapt software (Martin, 2011. *EMBnet Journal* 17:10-12) alignment with the reference genome was performed with help of the TopHat software (Trapnell, *Nature Protocols* 7:562, 2012), and RNA-seq quality and alignment was evaluated using the SeqSolve software (Integromics). Finally, gene expression levels were quantified with help of the Cufflinks/Cuffdiff bioinformatic applications run with the SeqSolve software (Trapnell, 2010. *Nat Biotechnol* 28:511-515), and expressed as FPKM (Fragments per kilobase of exon per million fragments mapped) (data submitted to Gene Expression Omnibus, accession number GSE88836).
