# Supplementary figures and images for: Involvement of Igf1r in Bronchiolar Epithelial Regeneration: Role during Repair Kinetics after Selective Club Cell Ablation
Source: PLoS One. 2016 Nov 18;11(11):e0166388. doi: 10.1371/journal.pone.0166388 (PMC5115747; doi:10.1371/journal.pone.0166388)

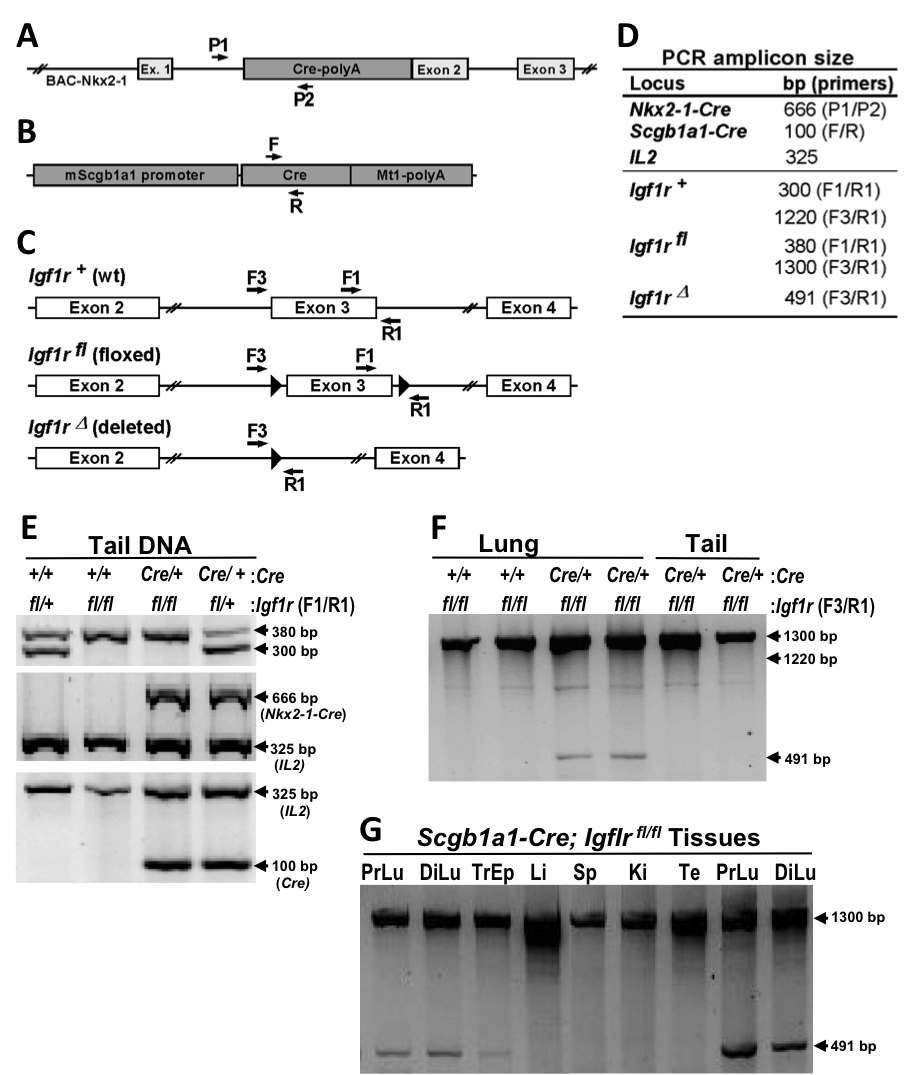

Supplement: S1 Fig — (A-B)Nkx2-1-Cre (A) and Scgb1a1-Cre (B) transgene elements and location of their respective primers (P1/P2 and F/R) for PCR genotyping. (C) Genomic DNA organization in alternative allelic forms of the Igf1r locus (wt, floxed and deleted), and specific primers (F1, F3 and R1) used for Igf1r locus analysis by PCR. (D) Expected amplicon sizes in PCR assays to identify the presence of Scgb1a1-Cre or Nkx2-1-Cre transgenes and the different Igf1r allelic forms. IL2 primers were used as constitutive positive controls when genotyping hemizygous Nkx2-1-Cre or Scgb1a1-Cre mice. (E) PCR mouse genotyping in tail DNA to identify Igf1r locus alleles (wt or floxed), and the presence of Cre transgenes using P1/P2 (for Nkx2-1-Cre) and F/R (for Scgb1a1-Cre), in combination with IL2 primers as an internal control. (F) PCR assays to determine the deleted allele of Igf1r (Δ) using F3/R1 primers on genomic DNA obtained from lung and tail of Igf1rfl/fl(+/+/fl/fl) as control mice, and Scgb1a1-Cre; Igf1rfl/fl(Cre/+/fl/fl) as mutant mice. The 491 bp fragment of the deleted form (Δ) is present only in lungs of Cre/+/fl/fl animals. (G) PCR assays of genomic DNA obtained from different tissues of Scgb1a1-Cre; Igf1rfl/fl. Note the presence of the deleted allele (Δ) in the tracheal epithelium (TrEp), and in the proximal (PrLu) and distal lung (DiLu) but not in the liver (Li), spleen (Sp), kidney (Ki), or testis (Te). bp, base pairs. (PNG) [file pone.0166388.s002.png]

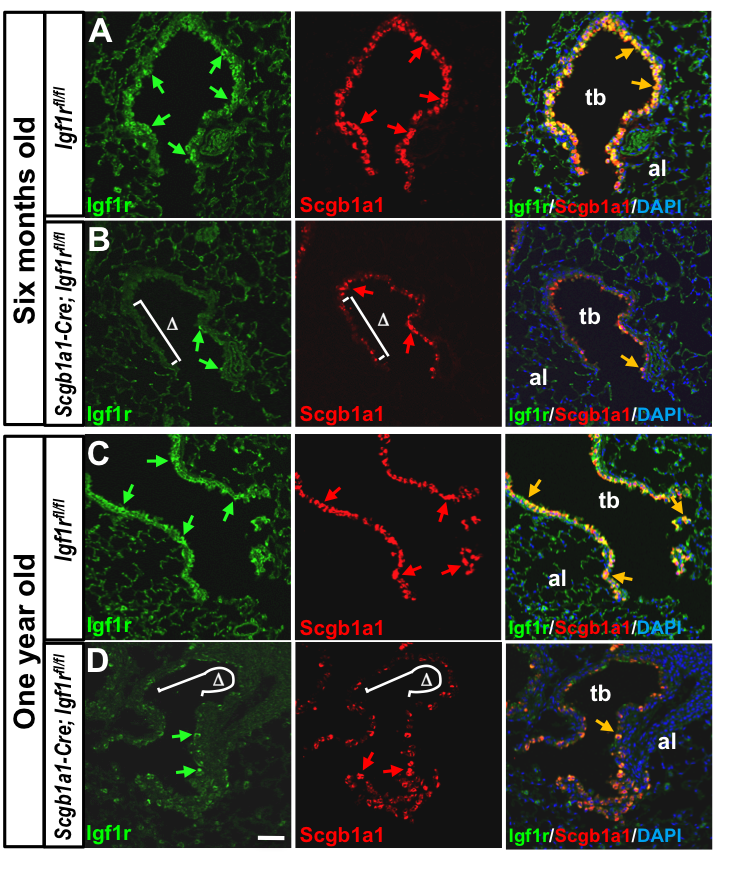

Supplement: S2 Fig — (A-D) Images of immuno-staining for Igf1r (green labeling in left panels) counter-stained with Scgb1a1 (red labeling in central panels) to identify club cells in terminal bronchioles, in lungs of Igf1rfl/fl (A, C) and Scgb1a1-Cre; Igf1rfl/fl (B, D) obtained from six months (A, B) and one year (C, D) old mice. Right panels are merged images of Igf1r/green (left panels) and Scgb1a1/red (central panels) to show co-localization of both markers in the club cells (orange), in addition to nuclear DAPI staining. Note that in control mice, Igf1r (green arrows, left panels in A and C) co-stained abundant Scgb1a1+ club cells (orange arrows, right panels in A and C). However, distal bronchiolar epithelium of Scgb1a1-Cre; Igf1rfl/fl mice show a strong reduction in the number of Igf1r+ (green arrows, left panels in B and D), sometimes organized in epithelial areas with complete lack of Igf1r expression (Δ). Lack of Igf1r correlated with a reduction in number and size of Scgb1a1+ club cells (central and right panels in B and D), and many of the remaining Scgb1a1+ epithelial cells, did not express Igf1r (colored in red, right panels in B and D). al, alveolus; tb, terminal bronchiole. Scale bar in D (left panel): 50 μm; applies to all panels. (PNG) [file pone.0166388.s003.png]

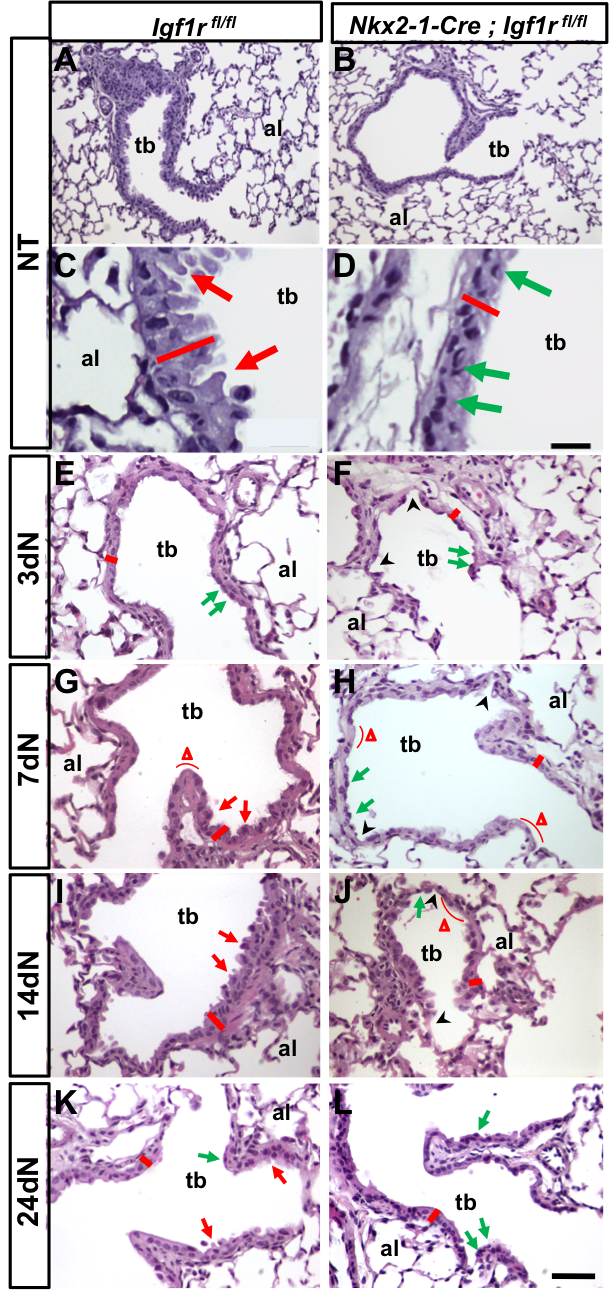

Supplement: S3 Fig — (A-D)Representative H&E staining of lung terminal bronchioles sections obtained from control Igf1rfl/fl (A, C) and mutant Nkx2-1-Cre; Igf1rfl/fl(B, D) three months old non-treated mice (NT) mice, at low (A-B) and high magnification (C-D). Note that the terminal bronchiolar epithelium of mutant mice shows epithelial flattening (red segment), thinner club cells, with absence of their cupulated shape (present in controls; red arrows in C), presence of aberrant ellipsoid nuclei (green arrows) (D) and interruptions in epithelial continuity (black arrowheads in F, G and J). (E-L)H&E staining of terminal bronchioles in control and mutant mice after naphthalene treatment at three (3dN)(E-F), seven (7dN)(G-H), fourteen (14dN)(I-J) and 24 (24dN)(K-L) days of recovery after challenge. Conditional mutant lungs show epithelial cells with ellipsoid nuclei protruding in the bronchiolar lumen (green arrows) and lack of club cells compared with the controls. Those observations are more evident at 7dN and 14dN where there are extensive areas with lack of “cupulated” club cells (Δ, red line). See morphological quantifications in Figs 3E and 5G–5E. al, alveolus; NT, no treatment; tb, terminal bronchiole. Scale bar in L: 50 μm in A-B, E-L. Scale bar in D: 10 μm in C-D. (PNG) [file pone.0166388.s004.png]

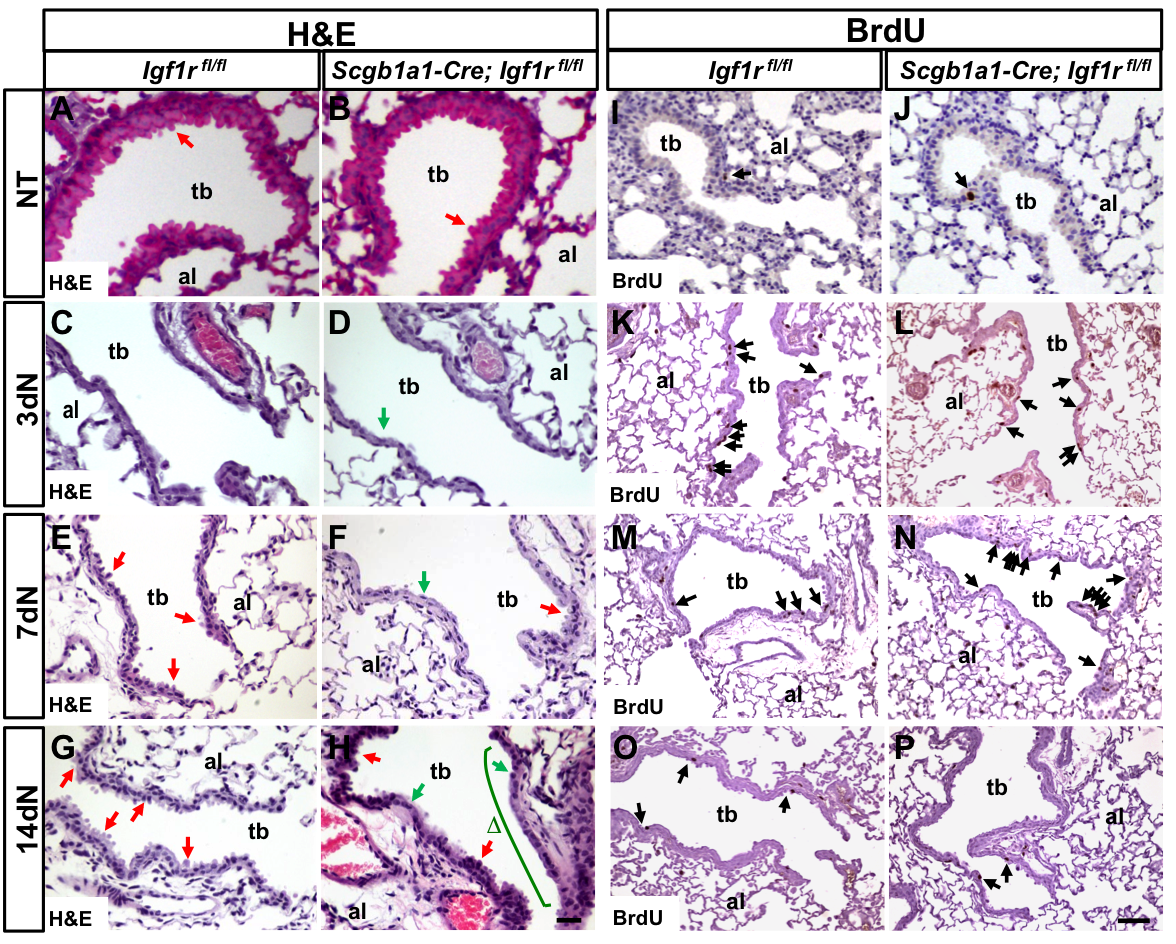

Supplement: S4 Fig — H&E histological (A-H) and BrdU immuno-histochemical (I-P) stainings to respectively evaluate the histology and proliferation in three months old control (Igf1rfl/fl) and mutant (Scgb1a1-Cre; Igf1rfl/fl) mice, either before (NT)(A-B; I-J) or after the naphthalene treatment at different stages of recovery: three (3dN)(C-D; K-L), seven (7dN)(E-F; M-N) and fourteen (14dN)(G-H; O-P) days. Note that the bronchiolar epithelium in mutant mice do not show evident histological alterations in club cells (B), compared with controls (A) (red arrows point to normal club cells). In terminal bronchioles of naphthalene treated mice, the Scgb1a1-Cre; Igf1r fl/fl mutant lungs show more club cells with altered morphology (green arrows) and less proportion of club cells (red arrows). At 14dN, extensive areas of the epithelium appear lacking protruding cupules of club cells (Δ, green line in H). After immuno-staining for BrdU (administered 2 h label prior sacrifice) the number of BrdU+ cells (labeled in brown, black arrows) in NT, 3dN and 14dN mice did not show evident differences between genotypes (I-J, K-L and O-P). However note the increased number of BrdU+ labeled at 7dN in the mutants (black arrows in N). al, alveolus; NT, no treatment; tb, terminal bronchiole. Scale bar in H: 20 μm in A-H. In P: 50 μm in I-P. (PNG) [file pone.0166388.s005.png]

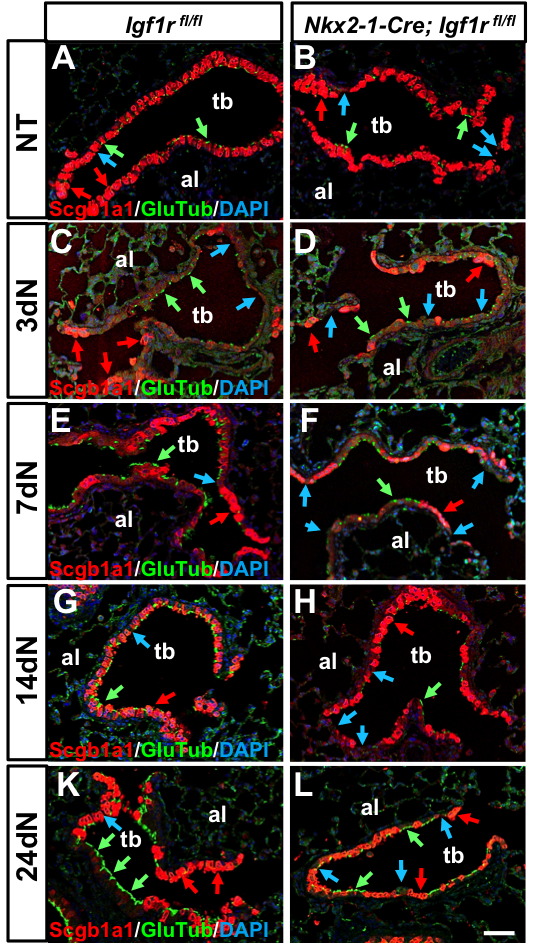

Supplement: S5 Fig — Representative images of immuno-staining to identify Scgb1a1+ cells in red, and ciliated/GluTub+ in green in lungs of Igf1rfl/fl (A, C, E, G, K) and Nkx2-1-Cre; Igf1rfl/fl (B, D, F, H, L) before (A-B) and after (C-L) naphthalene treatment. Counterstain with DAPI in blue label nuclei. Note the lower proportion of club (red arrows) and ciliated cells (green arrows), and the increased presence non-labeled epithelial cells (blue arrows) in Nkx2-1-Cre mutants. These phenotypes were more evident at 7dN and 14dN stages. See quantifications in Fig 6E. al, alveolus; NT, no treatment; tb, terminal bronchiole. Scale bar in L: 50 μm, applies to all panels. (PNG) [file pone.0166388.s006.png]

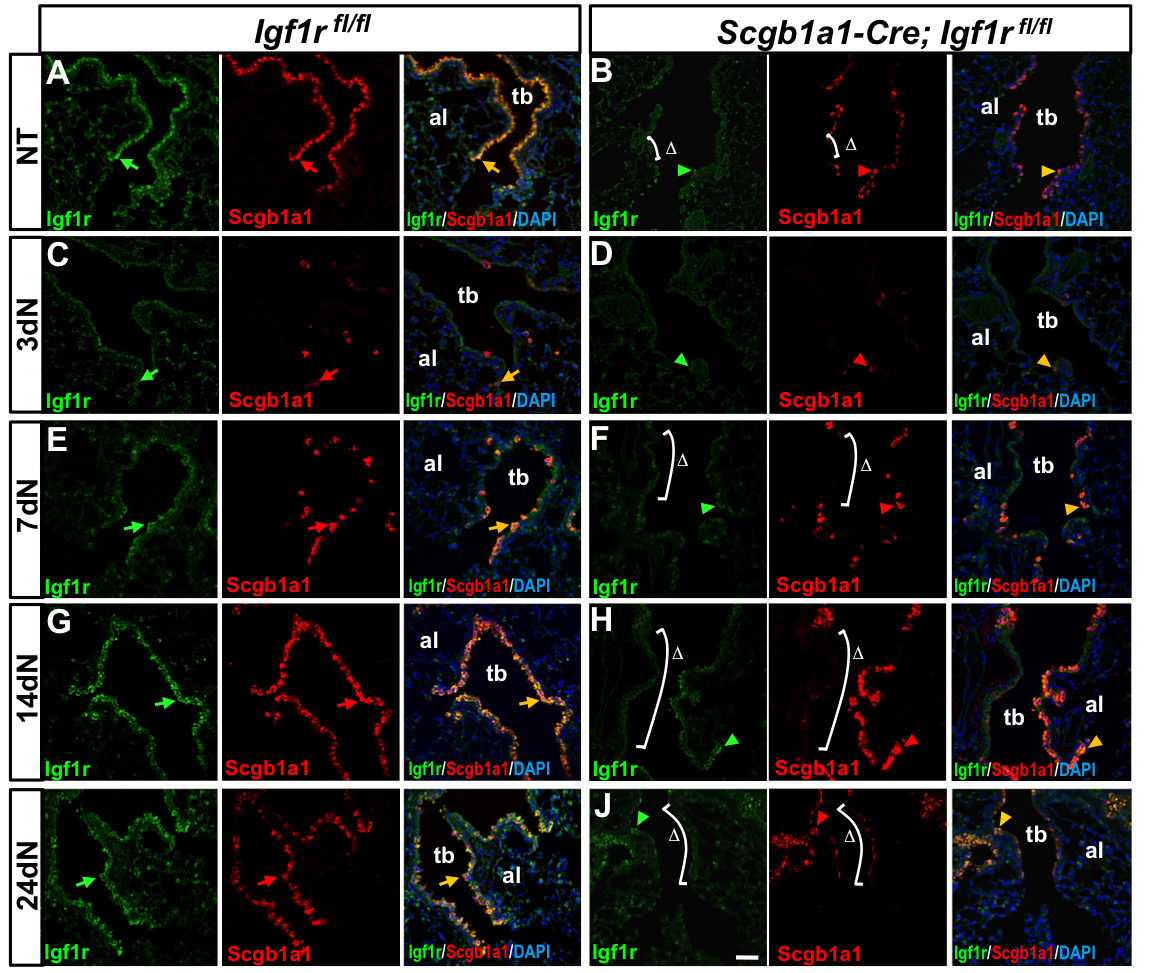

Supplement: S6 Fig — Immuno-staining for Igf1r (green labeling in left panels) and Scgb1a1 (red labeling in central panels) obtained from terminal bronchioles of Igf1rfl/fl (A, C, E, G, I) and Scgb1a1-Cre; Igf1rfl/fl(B, D, F, H, J) mice before (NT) and after different days of the naphthalene treatment (dN). Right panels are merged images of Igf1r/green (left panels), Scgb1a1/red (central panels) to show co-localization (orange) of both markers in club cells, in addition to nuclear DAPI staining. (A-B) Terminal bronchioles of NT mice. Note that in control mice, Igf1r (green arrow, left panel in A) co-stained abundant Scgb1a1+ club cells (orange arrow, right panel in A). However, distal bronchiolar epithelium of Scgb1a1-Cre; Igf1rfl/fl mice show scarce of Igf1r+/Scgb1a1+ cells (green, red and orange arrowheads in B), sometimes organized in epithelial areas with complete lack of Igf1r expression (Δ) (See S2 Fig). (C-J)Immuno-staining as described in A-B, after naphthalene treatment at three (3dN)(C-D), seven (7dN)(E-F), fourteen (14dN)(G-H) and twenty four (24dN)(I-J) days after the naphthalene challenge. Note the reduced or complete lack of Igf1r epithelial staining (Δ, white segments in left panels), the delayed regeneration in club cells (reduced proportion of red cells compared to controls, in central panels), and reduced numbers of cells, but still present, that retains Igf1r/Scgb1a1 co-expression (arrowheads). al, alveolus; NT, no treatment; tb, terminal bronchiole. Scale bar in J: 50 μm; applies to all panels. (PNG) [file pone.0166388.s007.png]

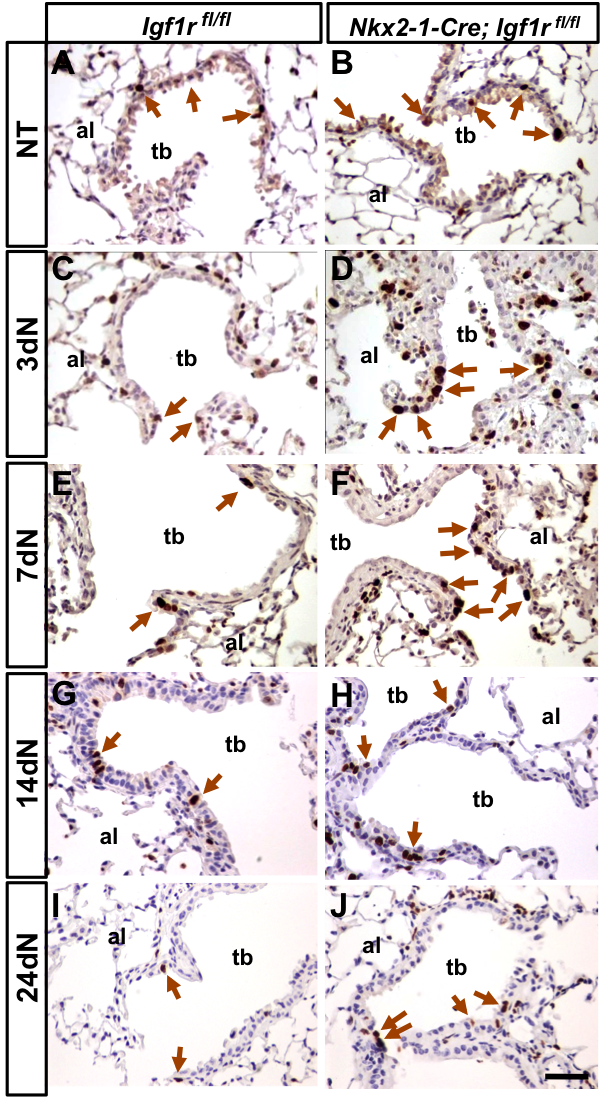

Supplement: S7 Fig — Representative images of Ki67 immunostaining (brown nuclei and arrows) in terminal bronchioles of Igf1rfl/fl (A, C, E, G, I) and Nkx2-1-Cre; Igf1rfl/fl (B, D, F, H, J) lungs before (NT)(A-B) and after three (3dN)(C-D), seven (7dN)(E-F), fourteen (14dN)(G-H) and twenty four (24dN)(I-J) days after the naphthalene treatment. Note the increased presence of Ki67+ cells in the epithelium of Nkx2-1-Cre mutant mice respect to controls, at all stages. See quantifications in Fig 7C. al, alveolus; NT, no treatment; tb, terminal bronchiole. Scale bar in J: 50 μm; applies to all panels. (PNG) [file pone.0166388.s008.png]

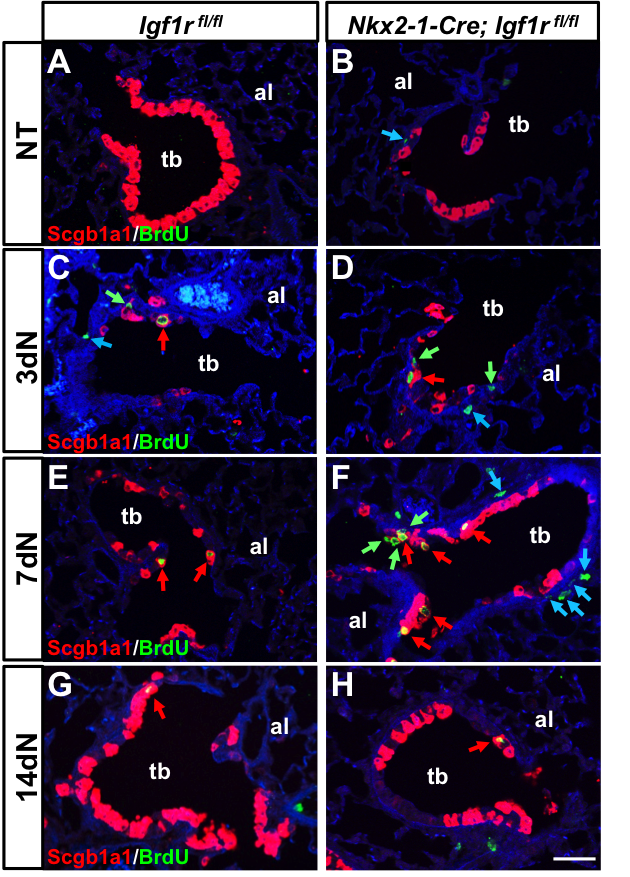

Supplement: S8 Fig — (A-H)Immuno-staining for BrdU labeling (green labeled nuclei) in terminal bronchioles of Igf1rfl/fl (A, C, E, G) and Nkx2-1-Cre; Igf1rfl/fl(B, D, F, H) mice before (A-B) and after three (3dN)(C-D), seven (7dN)(E-F) and fourteen (14dN)(G-H) days after the naphthalene treatment. Sections were co-stained with Scgb1a1 (red labeling) allowing to differentiate three groups of cells: total BrdU+ cells in the bronchiolar epithelium (green arrows), BrdU+/Scgb1a1+ double labeled cells (red arrows) and BrdU+ cells under the epithelial basal membrane (blue arrows). Note the increased number of bronchiolar epithelial cells proliferating in the mutants at 7dN (E-F) (green and red arrows), accompanied by an increased number of BrdU labeled cells below the basal membrane nearby the epithelium (blue arrows). See quantification in Fig 7H. al, alveolus; NT, no treatment; tb, terminal bronchiole. Scale bar in H: 50 μm; applies to all panels. (PNG) [file pone.0166388.s009.png]

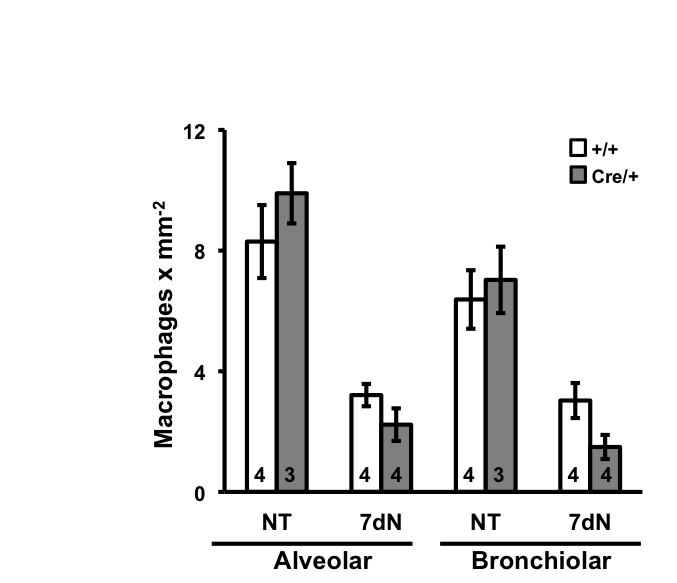

Supplement: S9 Fig — The graphic represents the quantification of F4/80 stained cells under the confocal microscope observation in alveolar and bronchiolar areas of control (+/+) and mutant (Cre/+) mice, in non-treated (NT) and at day seven (7dN) after the naphthalene treatment. Quantification of macrophages in the bronchiolar area corresponds to measurements performed in confocal picture frames of bronchiolar fields counting all F4/80+ cells, including those located in the surrounding alveolar parenchyma. Note the reduced presence of macrophages in both genotypes at 7dN, although without significant differences between genotypes. (PNG) [file pone.0166388.s010.png]
