# Supplementary material for: Involvement of Igf1r in Bronchiolar Epithelial Regeneration: Role during Repair Kinetics after Selective Club Cell Ablation
Source: PLoS One. 2016 Nov 18;11(11):e0166388. doi: 10.1371/journal.pone.0166388 (PMC5115747; doi:10.1371/journal.pone.0166388)
Supplement: S1 Table — (DOCX) [file pone.0166388.s011.docx]

**S1 Table**. Source and dilution of primary and secondary antibodies used in immunodetection: Immunohistochemistry (IHQ) and Western blotting.

| **Primary Antibodies (IHQ)** | | | | | **Dilution** | | **Manufacturer** | |  |
| --- | --- | --- | --- | --- | --- | --- | --- | --- | --- |
| Igf1 (RB-9240PO); Polyclonal, rabbit | | | | | 1:25 | | Lab Vision/Neomarkers | |  |
| Igf1rβ (C20) (Sc-713); Polyclonal, rabbit | | | | | 1:50 | | Santa Cruz Biotech. Inc. | |  |
| * Igf1rβ (3027); Polyclonal, rabbit | | | | | 1:50 | | Cell Signaling Technology, Inc. | | |
| * Igf1rβ (PA1746); Polyclonal, rabbit | | | | | 1:50 | | Boster Biological Technology Co. | | |
| Cgrp (N20) (Sc-8856); Polyclonal, goat | | | | | 1:200 | | Santa Cruz Biotech. Inc. | | |
| aSMA (Clone 1A4) (A5228); Monoclonal, mouse | | | | | 1:500 | | Sigma-Aldrich Co. | | |
| Scgb1a1 (T18) (Sc-9772); Polyclonal, goat | | | | | 1:400 | | Santa Cruz Biotech. Inc. | | |
| Detyrosinated a-Tub (GluTub)(AB3201); Polyclon., rabbit | | | | | 1:300 | | Merck Millipore | | |
| Pdpn (MA5-18054); Monoclonal, hamster | | | | | 1:2000 | | Thermo Fisher Scientific Inc. / Pierce | | |
| Sftpc (precursor) (M-20) (Sc-7706); Polyclonal, goat | | | | | 1:100 | | Santa Cruz Biotech. Inc. | | |
| F4/80 (MCA497GA); Monoclonal, rat | | | | | 1:100 | | AbD Serotec | | |
| Pecam1 (M20) (Sc-1506); Polyclonal, goat | | | | | 1:100 | | Santa Cruz Biotech. Inc. | | |
| Nkx2-1 (MAD-210486Q); Monoclonal, mouse | | | | | 1:100 | | Master Diagnóstica | | |
| BrdU [BU1/75 (ICR1)] (ab6326); Monoclonal, rat  p21 (M-19) (Sc-471); Polyclonal, rabbit | | | | | 1:200  1:50 | | Abcam  Santa Cruz Biotech. Inc. | | |
| Ki67 (MAD-020310Q); Monoclonal, rabbit | | | | | 1:200 | | Master Diagnóstica | | |
| **Primary Antibodies (Western blot)** | | | **Dilution** | | | | | **Manufacturer** |  |
| Scgb1a1 (T18) (Sc-9772); Polyclonal, goat  IGF-IRß (H60) (sc-9038); Polyclonal, rabbit | | | | | 1:2000  1:1000 | | Santa Cruz Biotech. Inc.  Santa Cruz Biotech. Inc. | |  |
| P-AKT1/2/3(ser 473) (sc-7985-R); Polyclonal, rabbit | | | | | 1:1000 | | Santa Cruz Biotech. Inc. | | |
| AKT1/2/3 (H-136) (sc-8312); Polyclonal, rabbit  P-p38MAPK (3D7) (9215); Monoclonal, rabbit  P38MAPK (9212); Polyclonal, rabbit  P-p44/42 MAPK (Erk1/2) (Tyr202/Tyr204) (197G2);  Monoclonal, rabbit  p44/42 MAPK(137F5) (4695); Monoclonal, rabbit  P-SAPK/JNK (Thr183/Tyr185) (98F2) (4671);  Monoclonal, rabbit  SAPK/JNK (56G8) (9258); Monoclonal, rabbit | | | | 1:1000  1:1000  1:1000    1:1000  1:1000    1:1000  1:1000 | | | Santa Cruz Biotech. Inc.  Cell Signaling Technology, Inc.  Cell Signaling Technology, Inc.    Cell Signaling Technology, Inc.  Cell Signaling Technology, Inc.    Cell Signaling Technology, Inc.  Cell Signaling Technology, Inc. | |  |
| **Secondary Antibodies (IHQ)** | | | **Dilution** | | | **Manufacturer** | | |  |
| Alexa Fluor 488® Chicken anti-rat | | | 1:1000 | | | Thermo Fisher Scientific Inc. / Invitrogen | | |  |
| Alexa Fluor 488® Goat anti-hamster | 1:500 | | | | Thermo Fisher Scientific Inc. / Invitrogen | | |  |  |
| Alexa Fluor 546® Donkey anti-rabbit | 1:500 | | | | Thermo Fisher Scientific Inc. / Invitrogen | | |  | |
| Alexa Fluor 633® Goat anti-mouse | 1:500 | | | | Thermo Fisher Scientific Inc. / Invitrogen | | |  | |
| Alexa Fluor 633® Donkey anti-goat | | | 1:400 | | | Thermo Fisher Scientific Inc. / Invitrogen | | |  |

* Igf1r antibodies sporadically used to corroborate data not included in Figures.
