# Supplementary material for: Involvement of Igf1r in Bronchiolar Epithelial Regeneration: Role during Repair Kinetics after Selective Club Cell Ablation
Source: PLoS One. 2016 Nov 18;11(11):e0166388. doi: 10.1371/journal.pone.0166388 (PMC5115747; doi:10.1371/journal.pone.0166388)
Supplement: S2 Table — (DOCX) [file pone.0166388.s012.docx]

**S2 Table**. List of primer sets used in qRT-PCR.

| ***Gene Symbol*** | ***GenBank***  ***Accession No.*** | ***Forward primer*** | ***Reverse Primer*** |
| --- | --- | --- | --- |
| ***18S*** | NR_003278.3 | 5’-ATGCTCTTAGCTGAGTGTCCCG-3’ | 5’-ATTCCTAGCTGCGGTATCCAGG-3’ |
| ***Igf1r*** | NM_010513 | 5’-CCAGAGCAAAGGGGACATAA-3’ | 5’-TGATTCGGTTCTTCCAGGTC-3’ |
| **** Igf1r*** | NM_010513 | 5’-CCGCTGCTGGACCACAA -3’ | 5’- CTCGCTTCCCGCACACA-3’ |
| ***Igf1*** | NM_010512 | 5’-cagaagcgatggggaaaat-3’ | 5’- gtgaaggtgagcaagcagag-3’ |
| ***Igf2*** | NM_010514 | 5’-ccttcgccttgtgctgcat-3’ | 5’-acggttggcacggcttgaa-3’ |
| ***Igf2r*** | NM_010515 | 5’-aggcaccaagatgaagcagt -3’ | 5’-aatgaaggggaagacacagg-3’ |
| ***Igfbp2*** | NM_008341 | 5’-gggagtgctggtgtgtga-3’ | 5’-ctgctggtgttcgggatg-3’ |
| ***Igfbp3*** | NM_008343 | 5’-gccctctgccttcttgattt-3’ | 5’-tcactcggttatgggtttcc-3’ |
| ***Igfbp4*** | NM_010517 | 5’-tgtgagattggattgtgtgtgt-3’ | 5’-tagagatggcgggataggag-3’ |
| ***Igfbp5*** | NM_010518 | 5’-GATGAGACAGGAATCCGAACAAG-3’ | 5’-AATCCT TTGCGGTCACAGTTG-3’ |
| ***Igfbp6*** | NM_008344 | 5’-AGGAGAGCAAACCCCAAGGA-3’ | 5’-TGAACAGGATTGGGCCGTATA -3’ |
| ***Insr*** | NM_01568 | 5’-TCCTGAAGGAGCTGGAGGAGT -3’ | 5’-CTTTCGGGATGGCCTGG -3’ |
| ***Scgb1a1*** | NM_011681 | 5’-ATGAAGATCGCCATCACAATCAC -3’ | 5’-GGATGCCACATAACCAGACTCT -3’ |
| ***Cyp2f2*** | NM_007817 | 5’-tgaggaaaatggaaggcaag -3’ | 5’-ccgaagacgacagagcagat -3’ |
| ***FoxJ1*** | NM_008021 | 5’-caggaggagcagggtgag -3’ | 5’-ggtagcagggcagttgatgt -3’ |
| ***Sfptc*** | NM_011359 | 5’-GAAGATGGCTCCAGAGAGCATC -3’ | 5’-GGACTCGGAACCAGTATCATGC -3’ |
| ***Aqp5*** | NM_009701 | 5’-GGTGGTCATGAATCGGTTCAGC -3’ | 5’-GTCCTCCTCTGGCTCATATGTG -3’ |
| ***Nkx2-1*** | NM_009385 | 5’-GCAGGTCAAGATCTGGTTCCAG -3’ | 5’-TGTCCTGCTGCAGTTGTTGCTG -3’ |
| ***Sox2*** | U_31967 | 5’-aaccaagacgctcatgaagaag -3’ | 5’-ctgcgagtaggacatgctgtag -3’ |
| ***Notch3*** | NM_008716 | 5’-GTGGTGATGCTGGAGATTGA-3’ | 5’-CCGAAGTGGGTATGGGAAA-3’ |
| ***Yap1*** | NM_001171147 | 5’-AGGAGAGACTGCGGTTGAAA-3’ | 5’-TTGCTGTGCTGGGATTGATA-3’ |
| ***FoxM1*** | NM_008021 | 5’- CCTGCTTACTGCCCTTTCCT -3’ | 5’-CAC ACCCATCTCCCTACACC -3’ |

**** Igf1r***: Pair of primers used to quantify *Igf1r* mRNA in Fig. 4.
